# Lung SPLUNC1 Peptide Derivatives in the Lipid Membrane Headgroup Kill Gram-Negative Planktonic and Biofilm Bacteria

**DOI:** 10.1021/acs.biomac.3c00218

**Published:** 2023-05-24

**Authors:** Tanvi Jakkampudi, Qiao Lin, Saheli Mitra, Aishwarya Vijai, Weiheng Qin, Ann Kang, Jespar Chen, Emma Ryan, Runxuan Wang, Yuqi Gong, Frank Heinrich, Junming Song, Yuan-Pu (Peter) Di, Stephanie Tristram-Nagle

**Affiliations:** † Biological Physics, Physics Department, Carnegie Mellon University, 5000 Forbes Avenue, Pittsburgh, Pennsylvania 15213, United States; ‡ Department of Environmental and Occupational Health, University of Pittsburgh, 130 DeSoto Street, Pittsburgh, Pennsylvania 15261, United States; § NIST Center for Neutron Research, National Institute of Standards and Technology, 100 Bureau Drive, Gaithersburg, Maryland 20899, United States

## Abstract

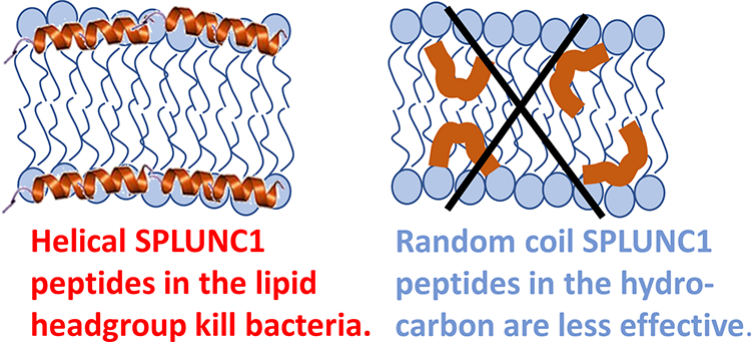

SPLUNC1 (short palate lung and nasal epithelial clone 1) is a multifunctional host defense protein found in human respiratory tract with antimicrobial properties. In this work, we compare the biological activities of four SPLUNC1 antimicrobial peptide (AMP) derivatives using paired clinical isolates of the Gram-negative (G(−)) bacteria *Klebsiella pneumoniae*, obtained from 11 patients with/without colistin resistance. Secondary structural studies were carried out to study interactions between the AMPs and lipid model membranes (LMMs) utilizing circular dichroism (CD). Two peptides were further characterized using X-ray diffuse scattering (XDS) and neutron reflectivity (NR). A4-153 displayed superior antibacterial activity in both G(−) planktonic cultures and biofilms. NR and XDS revealed that A4-153 (highest activity) is located primarily in membrane headgroups, while A4-198 (lowest activity) is located in hydrophobic interior. CD revealed that A4-153 is helical, while A4-198 has little helical character, demonstrating that helicity and efficacy are correlated in these SPLUNC1 AMPs.

## 1 Introduction

The increase in infections caused by multi-drug-resistant (MDR) bacteria has led to a global health crisis. These bacteria increase their planktonic titer and also form biofilms within the human body, leading to infections that are difficult to treat. Due to their clustered structure, biofilms are less treatable by antibiotics than planktonic bacteria. A group of MDR bacteria includes the ESKAPE pathogens (*Enterococcus faecium*, *Staphylococcus aureus*, *Klebsiella pneumoniae*, *Acinetobacter baumanii*, *Pseudomonas aeruginosa*, and *Enterobacter* species).^1^ ESKAPE pathogens use biofilm formation as a form of resistance and cause lethal diseases.^2^ A potential cure lies in antimicrobial peptides (AMPs).^3,4^ AMPs function primarily by perturbing the bacterial membrane through interactions with charged phospholipids on the membrane surface instead of perturbing a metabolic pathway.^5^ For this reason, they are much slower to cause bacterial resistance.^6^ The current work explores the potency of AMPs derived from human short palate lung and nasal epithelial clone 1 (SPLUNC1) protein.^7^ SPLUNC1, which is also referred to as bacterial-permeability-increasing fold containing family member A1 (BPIFA1), is a 256-amino acid protein involved in innate immunity of the human respiratory tract.^8^ It serves as a fluid-spreading surfactant, facilitating the clearance of mucus. Moreover, the protein binds to the main lipid, lipopolysaccharide, on the outer leaflet of the outer membrane of G(−) bacteria and possesses both bacteriostatic as well as antibiofilm properties.^9^ The human airway is continually exposed to airborne pathogens,^10^ yet respiratory infections are partially prevented because microbial organisms are regularly flushed by means of mucus clearance through the mucociliary apparatus (MCA).^11^ The MCA is composed of the airway surface liquid (ASL) and a variety of antimicrobial factors, including proteins and peptides. SPLUNC1 functions as a regulator of the ASL and provides the means for controlling mucociliary clearance of microbial organisms by regulating Na^+^ absorption.^12^

One domain within the SPLUNC1 secondary structure is α4, a 30-residue helical region on the SPLUNC1 protein.^13^ This motif exhibits a cationic amphipathic structure with a net charge of +2, which is similar to that of well-known innate AMPs such as LL-37 (net charge +6).^14^ Therefore, it was surmised that α4 could be the primary peptide with antimicrobial activity.^15^ However, it was found that the antimicrobial activity of α4 against *P. aeruginosa* was inefficient, so the cationic character of α4 was increased by adding lysine residues while retaining the number of hydrophobic residues.^15^ We have generated a shortened peptide with 24 residues (A4-short or A4S) displaying increased antibacterial activity.^2^ Using rational design, A4S’s primary structure was further modified to enhance its antimicrobial activity. The present work compares the efficiencies of four A4S derivatives (A4-153, A4-157, A4-183, A4-198) in treating infections in cell culture caused by MDR bacteria. The goal of this study was to compare the activities of these peptides against paired clinical isolates of bacterial strains from 11 patients, before and after treating them with colistin. The increasing clinical use of colistin has led to colistin resistance in G(−) bacteria.^16^ The pathway to colistin resistance in *Klebsiella pneumoniae* primarily involves Lipid A modification,^17^ and the timing of the COL-R mutations may be key to understanding this resistance.^18^ The A4S derivatives differ in length, charge, hydrophobic moment, and hydrophobicity. In order to understand the underlying mechanisms of the derivative peptides, we have carried out three biophysical techniques: circular dichroism (CD), X-ray diffuse scattering (XDS), and neutron reflectivity (NR), to probe the secondary structure and interactions between the A4S derivatives and LMMs of Gram-negative (G(−)) inner membranes. A second LMM is Euk33, which is the mimic of a typical eukaryotic membrane with 33 mole% cholesterol.

## 2 Experimental Section

### 2.1 Peptides

**Figure 1:**
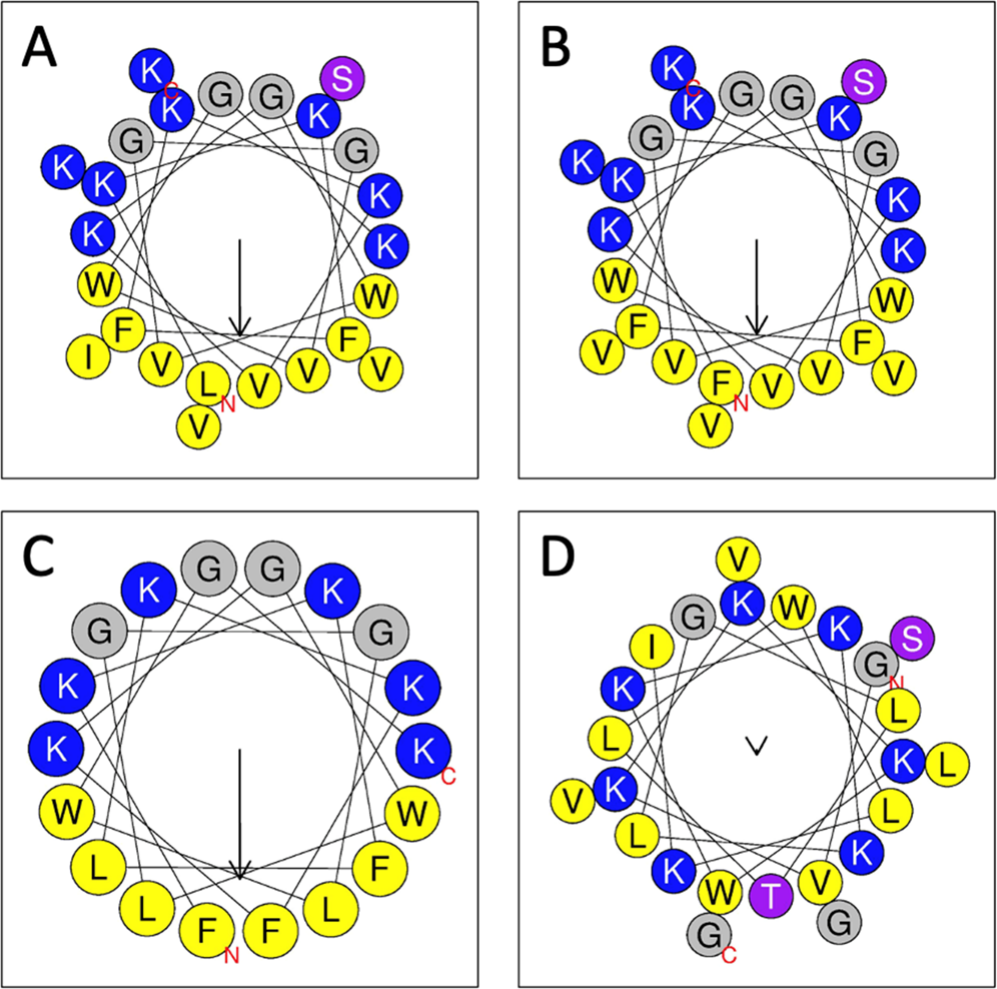
Helical wheel representations of (A) A4-153, (B) A4-157, (C) A4-183, and (D) A4-198. Arrows show the direction and relative strength of μH. Colors: hydrophobic amino acids, yellow; hydrophilic amino acids, blue; OH-containing amino acids, purple; and glycine, gray.

Peptides (A4-153, A4-157, A4-183, A4-198) were synthesized by Genscript (Piscataway, NJ) with HPLC/MS results shown in the S.I. (Figures S6–S9). Amino acid sequences are shown in Table 1; A4-198 is a version scrambled to α-helical content, with a similar hydrophobicity as A4-153. Peptide physical attributes are shown in Figure 1 and Table 1, where A4-198 has a very small hydrophobic moment (μH) due to the scrambling. In the helical wheel representation shown in Figure 1D, hydrophilic and hydrophobic residues are not separated, as shown in Figure 1A–C. Purity was 98%, as shown by mass spectroscopy analysis. In addition, colistin was used as a clinical antibiotic control of the methodology, and LL-37, the human cathelicidin, was also used as natural AMP control, since it is ineffective against the tested G(−) bacteria.

**Table 1:** Physical Attributes of Peptides

Sample	AA sequence	#AA residues	Z	μH	H	μH/H
A4-153	LKKFFKKVKGWVGGVWGKVKS	24	8	0.65	0.405	1.60
A4-157	FKKFFKKVKGWVGGVWGKVKS	24	8	0.64	0.385	1.66
A4-183	FKKFLKKFKGWLGGLWGK	18	6	0.70	0.502	1.40
A4-198	GVKKKWKKKLGLTLWLKISGVV	24	7	0.04	0.489	0.07

#### 2.1.1 Bacteria and Cells

The above peptides were tested against 25 substrains of *Klebsiella pneumoniae*. 24 substrains were clinical isolates obtained from the University of Pittsburgh Medical Center (UPMC), and one was a lab strain. The clinical substrains were A2-Obscure, **A5**, B2, **B3-Bright**, B5, **B6**, B8, **B9**, C2, **C3,** C4, **C5,** D4, **D7,** E5, **E6**, F2, **F3**, F8, **F9,** H4-Bright, **H5,** I1, **I2,** where the substrains in boldface represent resistance to colistin, and the substrains in regular typeface signify initial susceptibility to colistin prior to these experiments. One pair, B3-Bright, did not show the expected colistin-resistant/sensitive characteristics, so we decided to remove them from the analysis. Murine immortal cells, RAW 264.7 and 3T3 fibroblast, as well as two additional cell lines, were used for the eukaryotic toxicity studies. They were obtained from the American Type Culture Collection (ATCC). The BioLegend (San Diego, CA) TetraZ cell counting kit was used to determine viable cell count.

#### 2.1.2 Biological Reagents

Dulbecco’s modified Eagle’s medium (DMEM) and Penicillin–Streptomycin (P/S) were purchased from Invitrogen (Carlsbad, CA). Other reagents were purchased from Sigma-Aldrich (St. Louis, MO): Mueller-Hinton Broth 2 (MHB2), phosphate-buffered saline (PBS), fetal bovine serum (FBS), and dimethyl sulfoxide (DMSO). Crystal violet was purchased from VWR International (Franklin, MA).

#### 2.1.3 Biophysical Reagents

**Figure 2:**
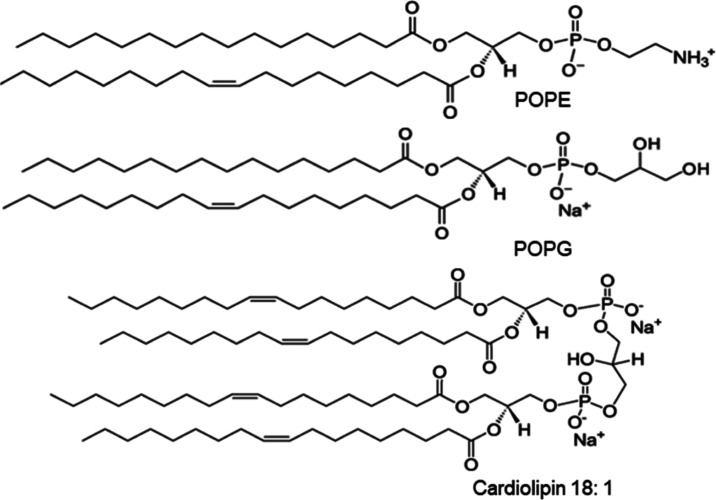
G(−)IM lipid model membrane composition: POPE/POPG/Cardiolipin 18:1, (7:2:1) molar ratio. Chemical structures from Avanti Polar Lipids.

Synthetic lipids were purchased from Avanti Polar Lipids (Alabaster, AL). The synthetic lyophilized lipids 1-palmitoyl-2-oleoyl-*sn*-glycero-3-phosphoethanolamine (POPE), 1-palmitoyl-2-oleoyl-*sn*-glycero-3-phospho-(10-rac-glycerol) sodium salt (POPG) and 10,30-bis-[1,2-dioleoyl-*sn*-glycero-3-phospho]-*sn*-glycerol sodium salt (TOCL, i.e., cardiolipin 18:1), 1-palmitoyl-2-oleoyl-*sn*-glycero-3-phosphocholine (POPC), and cholesterol were used as received. Lipid chemical structures are shown in Figure 2. Organic solvents were HPLC grade from Sigma-Aldrich (St. Louis, MO). Dulbecco’s phosphate-buffered saline (PBS) buffer was purchased from Sigma-Aldrich (St. Louis, MO) and diluted 1:10 with Milli-Q water, since 150 mM PBS has significant ellipticity. Lipid stock solutions were combined to create lipid mixtures in molar ratios mimicking the Gram-negative inner membrane G(−)(IM), POPE/POPG/TOCL (7: 2: 1). Average lipid composition of the bacterial membrane model was based on ref (19). A eukaryotic LMM typical of a white blood cell was composed of POPC/POPE/Chol (5:1:3) molar ratio.

### 2.2 Antibacterial Assays

Minimum inhibitory concentration (MIC, defined as the minimum AMP concentration to prevent bacterial growth) and biofilm data were obtained by UV–vis spectroscopy using a BioTek Epoch 2 microplate reader.

#### 2.2.1 Planktonic

*K. pneumoniae* substrains were grown overnight using 50 mL plastic conical tubes on a shaker platform at 37 °C. The following day, serial dilutions of the four SPLUNC1 peptides and LL-37 were tested against these 25 substrains of *K. pneumoniae* using a 96-well plate. The highest peptide concentration tested was 32 μM; if the bacteria still grew at this concentration, it was assumed that its minimum inhibitory concentration (MIC) value was 64 μM in order to determine average MIC values. Following the addition of the peptides at the tested concentrations from serial dilutions to the *K. pneumoniae* substrains, the plates were incubated at 37 °C for 16 h. Then, visible spectroscopy at λ = 570 nm was performed to determine the bacterial absorption in each well. Those wells with an optical density reading above a threshold value were deemed resistant to the peptide, while those that were below the threshold value were deemed sensitive to the peptide. The threshold value was determined using Gen5 software with the Di lab Growth Inhibition Assay protocol.

#### 2.2.2 Statistics

Average MICs and standard deviations were calculated by first averaging the absorbances as a function of AMP concentration in all of the bacterial strains from four total data sets. Then, the average MICs were determined from the averaged absorbances. The standard deviations of the MICs were calculated from each of the absorbances that were used to calculate the average. Then, the standard deviations were averaged across substrains for the graph. These procedures were used to obtain an average MIC with standard deviation across all 25 substrains for one peptide, in order to compare peptide efficacy. Typically, many more substrains are used to obtain an average MIC, so this average MIC may change if thousands of substrains are compared.

#### 2.2.3 Biofilms

The planktonic samples in the wells were then drained by inversion of the plate. The plate was washed with PBS prior to staining with 125 μL of 0.5% Crystal Violet (in 20% ethanol) for 15 min. Excess stain was removed by washing twice with distilled water. The bacterial remnants on the walls of the wells were analyzed at λ = 620 nm to observe any adhered bacteria. The absorbance values as percent of control (no AMP) do show a tendency of bacterial adherence to the wells. In this work, we will refer to the adhered bacteria after incubation at 37 °C for 16 h as biofilms.

### 2.3 Cell Killing Assays

The TetraZ cell counting kit was used to quantify cell proliferation and cell viability. It is based on a water-soluble tetrazolium salt, which, when reduced by cellular dehydrogenases, produces a chromophore. ∼5 × 10^4^ immortalized mouse RAW 264.7 macrophages and 3T3 fibroblast cells were incubated in a 96-well plate and allowed to attach for 24 h. AMPs in cell culture medium (DMEM D5796, 10% FBS, 1% P/S) were added to the wells. Cells were incubated at 37 °C for 4 h with the SPLUNC1 peptide derivatives at 8, 16, 32, and 64 μM concentrations. 10 μL of TetraZ was added and incubated for an additional 1.5 h at 37 °C. A Biotek Epoch 2 visible spectrophotometer was used to read the absorbance. The absorbance by the chromophore at λ = 450nm is proportional to the number of live cells (see standard curves, Figures S1 and S2, and additional cell line results (Figures S3 and S4) in the S.I.).

### 2.4 Circular Dichroism (CD)

Unilamellar vesicles (ULVs) of ∼600 Å diameter were prepared using an Avanti extruder: 200 μL of 20 mg/ml G(−)IM lipid in 15 mM PBS was extruded 21 times through Nucleopore filters of size 500 Å using 0.2 mL Hamilton syringes in the Avanti extruder. The final concentration of G(−)IM lipid in the ULVs was determined gravimetrically to be 15 mg/ml. Concentrated ULVs were added to 3 mL of 10 μM peptide in 15 mM PBS at pH 7 to create lipid/peptide molar ratios between 0:1 and 70:1. The samples remained at room temperature for ∼16 h before the CD measurement. Data were collected in 3 mL quartz cuvettes using a Jasco 1500 CD spectrophotometer at 37 °C in the Center for Nucleic Acids Science and Technology at Carnegie Mellon University. The samples were scanned from 200 to 240 nm 20 times, and the ellipticity (ϵ) results were averaged. Temperature was controlled at 37 °C via a Peltier element and water circulation through the sample compartment. Nitrogen gas was used at a flow rate between 20 and 25 ft^3^/h (CFH). The parameters for scanning were: speed 100 nm/min, step size 1.0 nm, response time 1 second, bandwidth 1 nm, and sensitivity of 20 mdeg. OriginPro 2019 (OriginLab, Northampton, MA) was used to carry out a linear least-squares fit of the smoothed ellipticity traces to four secondary structural motifs representing α-helix, β-sheet, β-turn, and random coil.^20^ This analysis gives a percentage match of each of the secondary structural motifs to the total sample ellipticity. Data were finally converted to mean residue ellipticity, taking into account the peptide concentration (10 μMolar) and number of amino acids (*N*), using the equation: MRE = (10^4^/*N*)ϵ deg cm^2^/dmol.

### 2.5 X-ray Diffuse Scattering (XDS)

Model membranes were prepared using the Rock and Roll procedure,^21^ which mixes 4 mg of lipids and peptides in organic solvent (trifluoroethanol/ chloroform 2:1 v-v), plates them onto chromic acid-cleaned silicon wafers (1 × 15 × 30 mm^3^) producing ∼1800 oriented bilayers in a stack, and then dries the wafers under vacuum for at least 2 h. Lipid/peptide molar ratios varied from 1000:1 to 75:1. Samples were fully hydrated in a thick-walled hydration chamber with mylar windows for X-rays.^22^ XDS data were collected at the Cornell High Energy Synchrotron Source (CHESS), Ithaca, NY, using wavelengths 0.8434 and 1.0976 Å, and at the home source using a Rigaku RUH3R (Tokyo, Japan) rotating anode generator with X-ray wavelength 1.5418 Å. All samples were measured at 37 °C. The XDS data are analyzed using liquid crystal theory with methods described in detail in the S.I. to ref (23). Full hydration causes the membrane stacks to fluctuate, producing lobes of diffuse data,^24,25^ which provide the intensities that are the basis for the form factors. Taking the Fourier transform of the form factors using the Scattering Density Profile modeling approach^26^ yields the electron density profile, which gives structural quantities.^22,26,27^

### 2.6 Neutron Reflectivity (NR)

6 mg of G(−)IM lipid/peptide mixtures were cosolubilized in organic solvent, dried under vacuum, and hydrated for 1–2 h via bath sonication in 1.2 mL of 2M NaCl. A single membrane bilayer was deposited onto a lipid-tethered gold-covered 3″ silicon wafer over ∼2 h using the vesicle fusion method.^28^ NR data were collected at the NG-D-MAGIK reflectometer^29^ at the NIST Center for Neutron Research (Gaithersburg, MD) over a momentum transfer range 0–0.25 Å^–1^. 6-h scans were collected in either H_2_O or D_2_O at 37 °C. Data were analyzed at NIST; 1D-structural profiles were parameterized using a continuous distribution model^30^ using Refl1D software. The component volume occupancy profile of the protein was defined by a Hermite spline with control points on average 15 Å apart. A Monte Carlo Markov Chain-based global optimizer was used to determine fit parameter confidence limits.^31^

## 3 Results

### 3.1 MIC in Planktonic Culture

**Figure 3:**
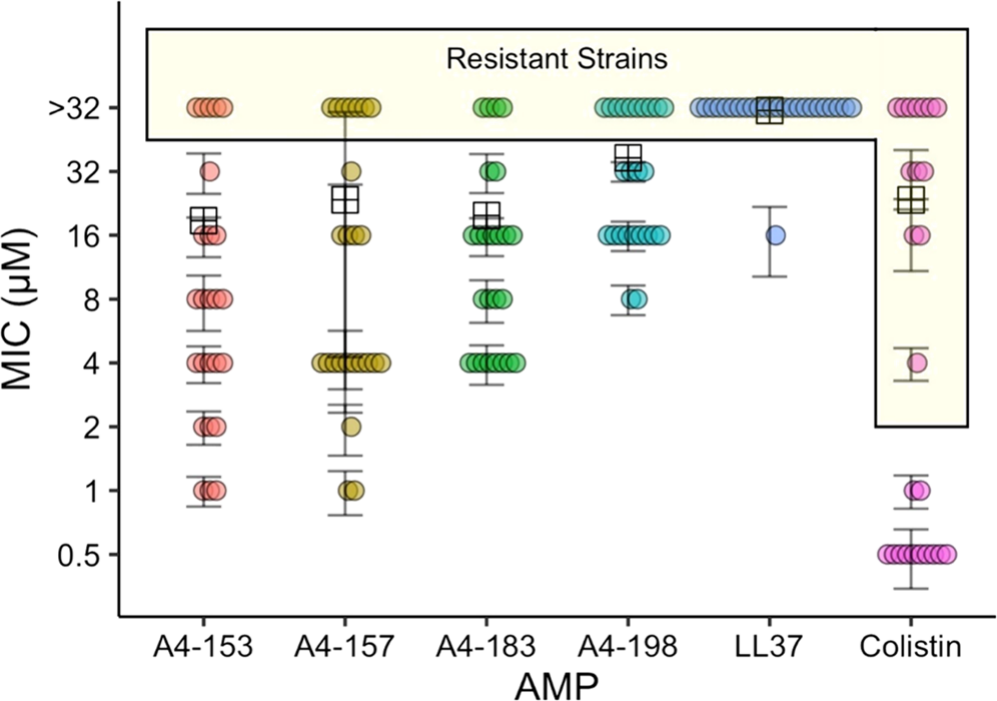
Mean MIC values of SPLUNC1-derived antimicrobial peptides, LL-37, and colistin for 24 clinical *K. pneumoniae* isolates and 1 lab strain with standard deviations. Data were collected in quadruplicate or duplicate. Graphical representation of MIC values, where data points inside the yellow box indicate resistant substrains above a threshold, while data points below the yellow box indicate bacterial sensitivity.

We measured the MIC values of four novel antimicrobial peptides (A4-153, A4-157, A4-183, and A4-198) as well as the human innate peptide LL-37 and the clinical AMP colistin when in planktonic culture of 24 clinical (patient-derived) *K. pneumoniae* isolates and 1 lab strain. The results are shown in Figure 3, where each data point represents one of the 25 bacterial substrains. The yellow-shaded box in Figure 3 highlights the AMP concentration at which the bacterial strains are resistant to AMP. We considered the bacteria to be resistant at AMP concentration > 32 μM, except for colistin, which has a resistance breakpoint at MIC value of 2 μM. Black crosses in boxes indicate the average MIC value for each AMP. As shown, A4-153 had the lowest average MIC value (18 μM), indicating that it is the most efficient of the SPLUNC1 AMPs. A4-183 had the next best efficiency (20 μM), followed by A4-157. Although the average MIC value for colistin is shown to be fairly high, this is due to the experimental design of using 11 colistin-resistant substrains and 13 colistin-susceptible substrains. Considering only the colistin-susceptible substrains, the average MIC of colistin is fairly low (0.88 μM). A4-198 was designed to test the hypothesis that low hydrophobic moment correlates negatively with efficacy (see Table 1). As shown in Figure 3, A4-198 has the highest MIC value. Averages of all of the substrains tested for each peptide are shown as black crosshatches.

### 3.2 Biofilm Growth

**Figure 4:**
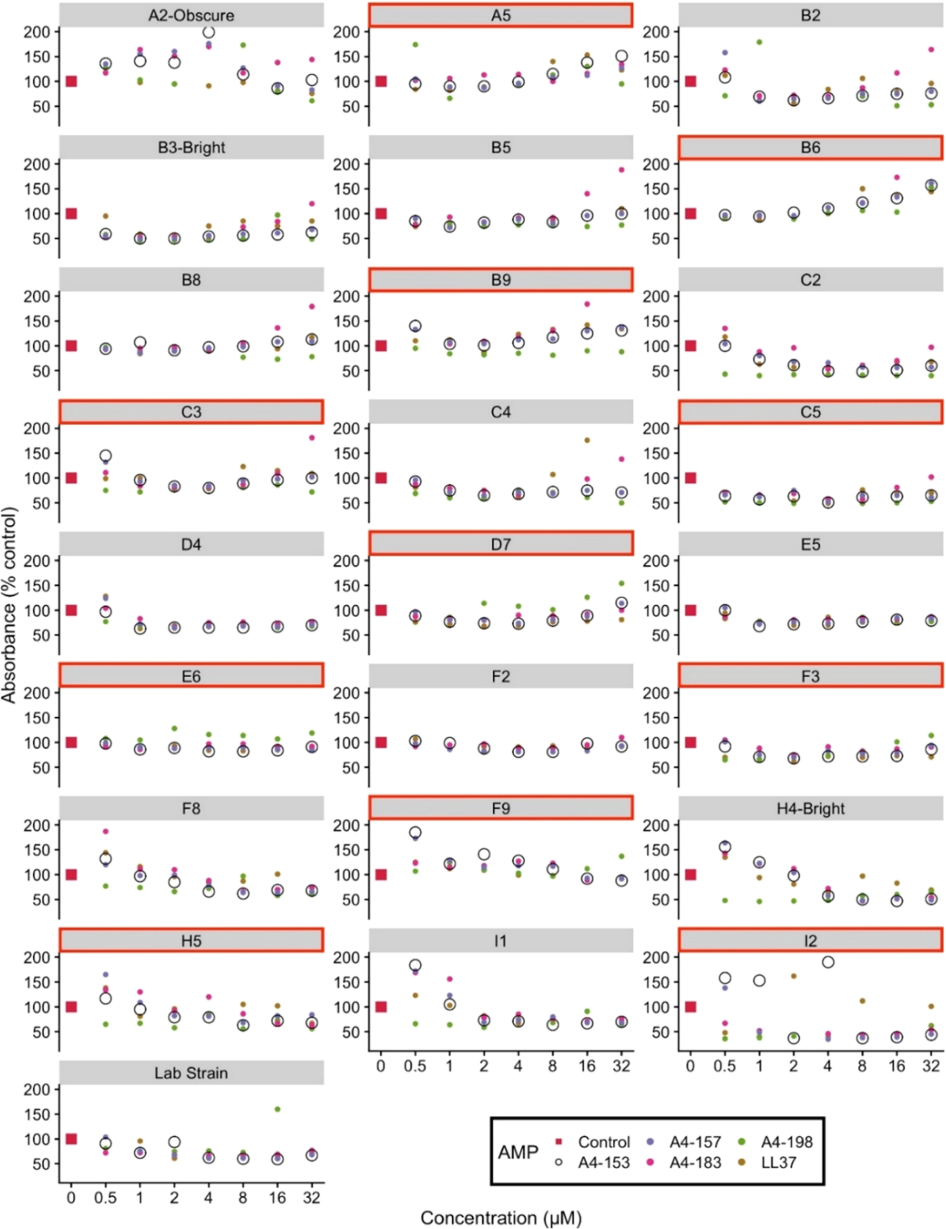
Biofilm growth of 24 clinical *K. pneumoniae* isolates (names of substrains shown on graphs) and 1 lab strain that were treated with SPLUNC1-derived AMPs and human LL-37. Each graph represents biofilm formation of one of the bacterial substrains. Red outlines indicate colistin-resistant strains. A4-153 is represented by open black circles to distinguish it from the other peptides. Most data were collected in quadruplicate, and a few in duplicate. The average standard deviation of the absorbance values is 15%.

We report the effects of these peptides on aggregated, immobile biofilm formation of the 25 strains. Upon draining the planktonic samples by inversion after 16 h, the biofilm bacteria that were adhered to the walls of the wells remained. These results are shown in Figure 4, in which A4-153 is represented by large, black open circles, while the other peptides are represented by smaller, colored solid circles. This distinction was made since the planktonic MIC values in Figure 3 indicated that A4-153 is the most successful at killing bacteria. In most of the 25 substrains shown in Figure 4, A4-153 displayed greater bacterial killing activity than the other peptides, although not at every concentration. For every substrain except A2-Obscure, B5, B8, B9, C2, C3, and C5, there existed a concentration range within which A4-153 was superior at preventing biofilm formation compared to the other A4S derivatives. This indicates that A4-153 is generally more efficient than the other peptides at preventing biofilm formation.

### 3.3 Eukaryotic Toxicity

**Figure 5:**
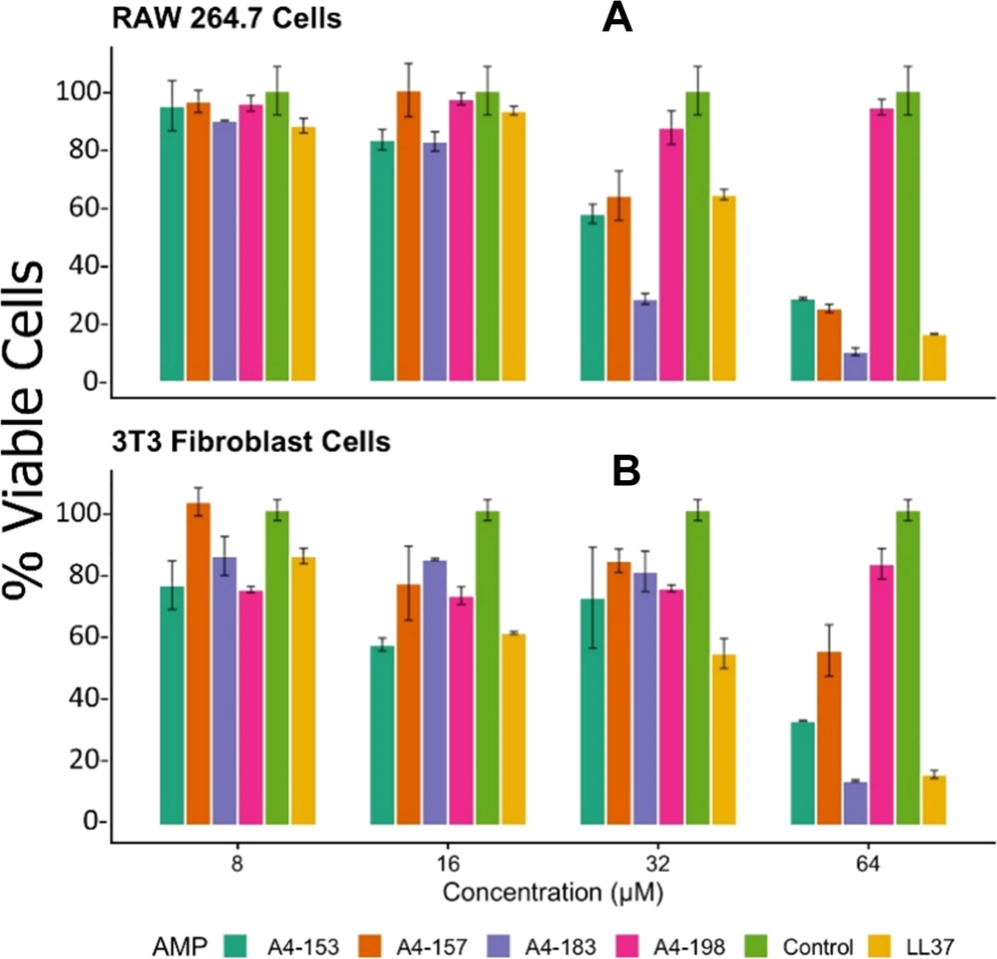
Cytotoxicity results of SPLUNC1 derivatives with two different eukaryotic cell types, RAW 264.7 cells and 3T3 fibroblasts. Error bars represent the standard error of duplicates. (A) Results for murine RAW 264.7 cells. (B) Results for murine 3T3 fibroblast cells. Data were collected in duplicate.

As shown in Figure 5A, at low concentration (8 μM), the A4S derivatives show no cytotoxicity as the % viable cells are nearly the same as in the control (no peptide). Apart from A4-198, all of the A4S derivatives display cytotoxic effects as a function of their increasing concentration in cell culture. In particular, at the highest concentration (64 μM), A4-183 has the greatest cytotoxic effect on the RAW 264.7 cells (Figure 5A), followed closely by LL-37. In Figure 5B in 3T3 fibroblast cells, at 8 μM, the cytotoxicities of A4-153 are comparable to A4-198, and slightly higher than the control. Additionally, at the highest concentration (64 μM), A4-198 has the lowest toxicity, while A4-153 shows ∼30% viable cells compared to the control. We estimate that 128 μM A4-153 would be required for 0% viable cells. Standard curves used to determine toxicity are shown in Figures S1 and S2. Additional toxicity studies using cell lines HBE and THP-1 (Figures S3 and S4) confirmed the general trend of Figure 5, but A4-183 was less toxic in THP-1 cells than in the other cell types.

### 3.4 Circular Dichroism (CD)

**Figure 6:**
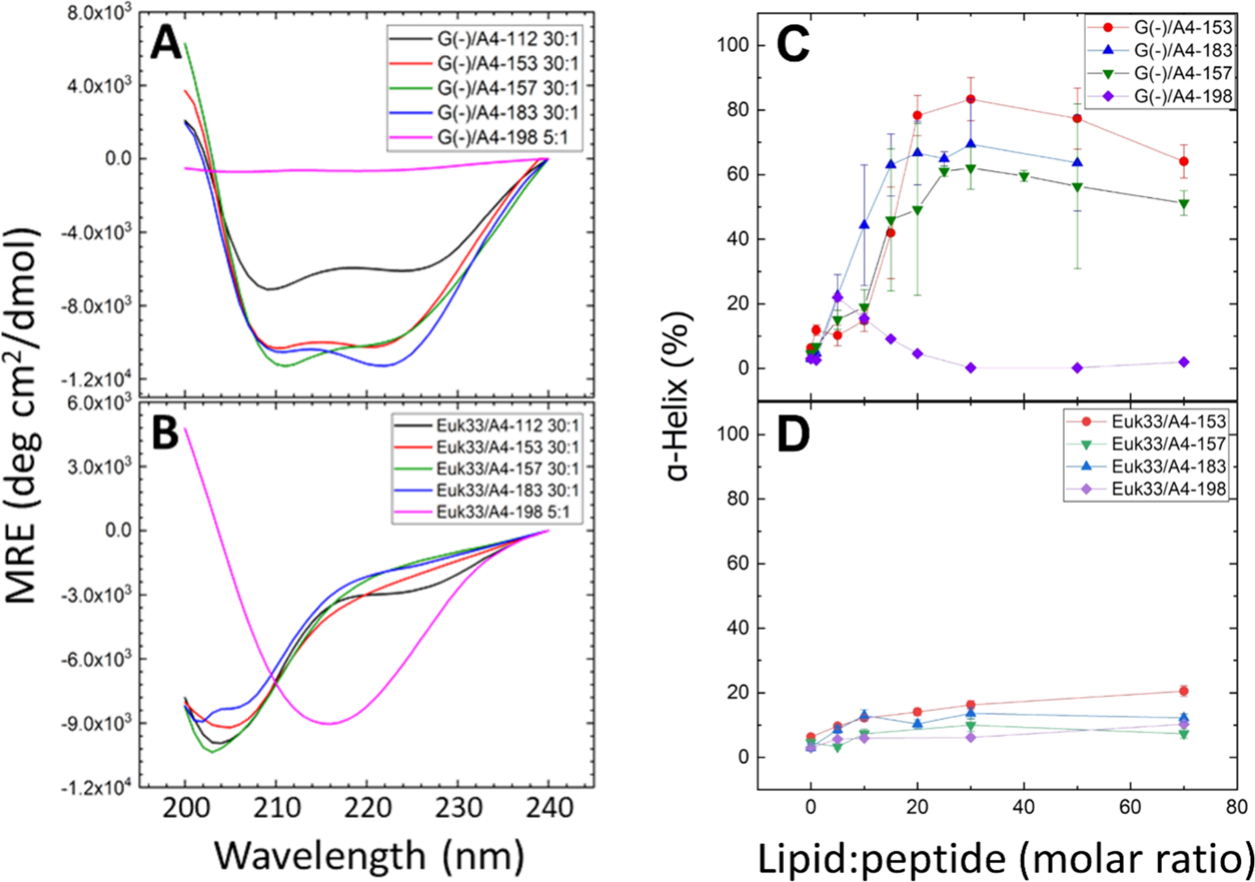
Mean residue ellipticity for (A) G(−)IM ULVs, (B) Euk33 ULVs. α-helical percentage vs lipid/peptide molar ratio for peptides in (C) G(−) or (D) Euk33 LMMs. Standard deviations in panels (C) and (D) were calculated from multiple fits to the data.

Mean residue ellipticity (MRE) results from spectroscopic scans of the SPLUNC1-derived peptides (A4-153, A4-157, A4-183, and A4-198) in unilamellar vesicles are shown in Figure 6A,B. A least-squares fitting procedure was applied to the 5-pts smoothed ellipticity traces in order to fit four secondary structural motifs (α-helix, β-sheet, β-turn, and random coil). The fit provides percent characterization of the secondary structures of each of these peptides. A summary of the α-helical content of these peptides is shown in Table 2, and summaries of all of the secondary structural motifs are shown in Tables S1–S8 in the S.I.

**Table 2:** Helical Content of SPLUNC1 Peptides

(a) G(−)		(b) EUK33	
G(−)/peptide molar ratio	α-helix (%)	Euk33/peptide molar ratio	α-helix (%)
A4-153 30:1	84	A4-153 30:1	16
A4-157 30:1	62	A4-157 30:1	10
A4-183 30:1	69	A4-183 30:1	14
A4-198 5:1	22	A4-198 30:1	6

In Table 2 and Figure 6A,B, the molar ratio of lipid/peptide of 30:1 was chosen for most of the peptides to compare their secondary structures directly. However, the maximum α-helical content varied as a function of the molar ratio, as shown in Figure 6C, D, where the maximum helical content did not always occur at 30:1 molar ratio. In contrast to the other peptides, A4-198 displayed very little α-helical content in all membrane mimics. In particular, the 5:1 molar ratio for A4-198 in Figure 6B was chosen for comparison because the α-helical content of A4-198 increased to a maximum at 5:1 molar ratio in G(−) inner membrane LMM.

### 3.5 X-ray Diffuse Scattering (XDS)

**Figure 7:**
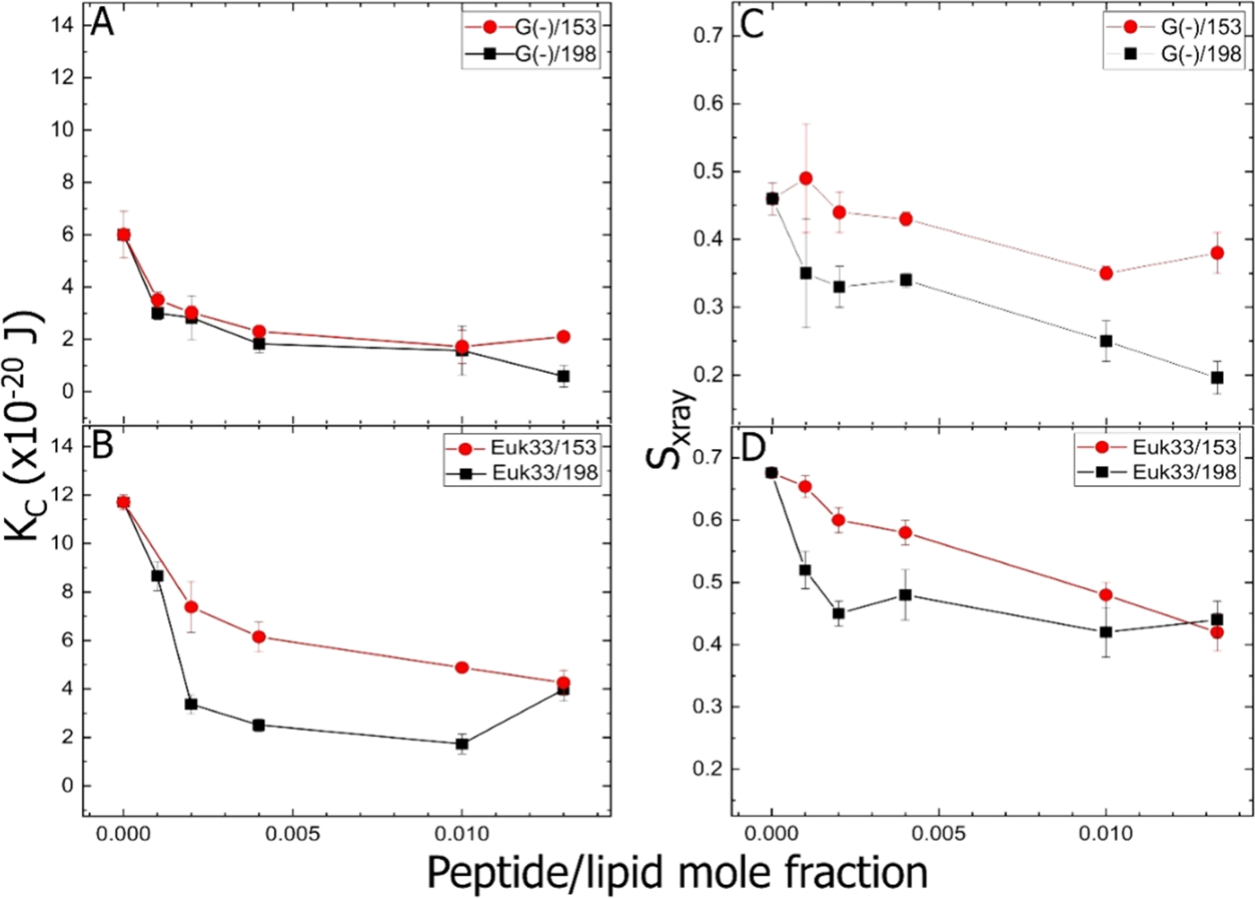
Elasticity results (*K*_C_, bending modulus) of A4-153 (red solid circles) and A4-198 (black solid squares). (A) G(−)IM LMMs, (B) Euk33 LMMs. Chain order parameter (*S*_xray_) of A4-153 and A4-198. (C) G(−)IM LMMs, (D) Euk33 LMMs. All are plotted as a function of peptide/lipid mole fraction. Standard deviations were calculated from duplicate or triplicate samples.

XDS was employed to determine the bending modulus *K*_C_ of the A4-153-containing G(−)IM model membranes. A4-153 was chosen for the XDS studies since it was the most efficient AMP according to MIC values, and A4-198 was chosen to compare to an inefficient peptide with the same number of amino acids. Higher *K*_C_ values indicate stiffening of the membrane, whereas lower *K*_C_ values indicate membrane softening. The XDS data shown in Figure 7A show a general softening for both A4-153 and A4-198 in G(−) LMM, with a slight stiffening at the highest A4-153 concentration. This small difference in membrane elasticity suggests that membrane mechanical properties are not correlated with G(−) bacterial killing.

Perhaps more important is their different chain order parameters, where Figure 7C shows that A4-153 has acyl chains that are more ordered with higher *S*_xray_ values than in the scrambled peptide A4-198 in G(−)IM LMMs. *S*_xray_ monitors the chain–chain correlation in a fluid phase bilayer. Figure 7B shows elasticity results in Euk33 LMMs. *K*_C_ decreased dramatically due to A4-198, and less so due to A4-153, but both peptides softened the Euk33 membrane. Acyl chain disordering for A4-153 and A4-198 in Euk33 LMMs paralleled the *K*_C_ behavior.

**Figure 8:**
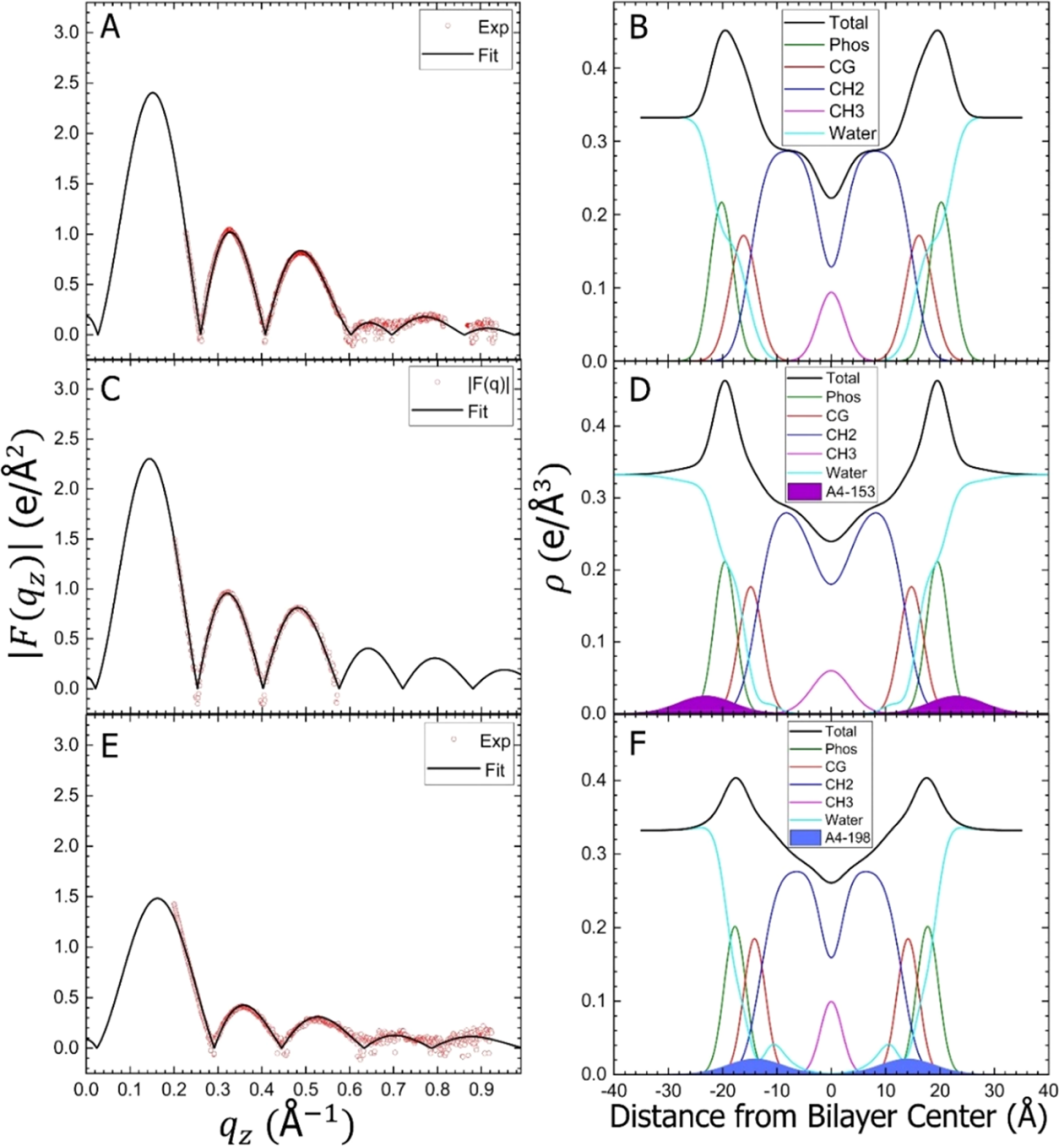
Form factor and EDP results for G(−)IM membranes. Form factors: (A) G(−) IM control, without peptide, (C) G(−)IM/A4-153 (75:1 molar ratio), and (E) G(−)IM/A4-198 (75:1). EDPs: (B) Control, (D) G(−)IM/A4-153 (75:1), and (F) G(−)IM/A4-198 (75:1). Component groups: phosphate + external headgroup (green), carbonyl–glycerol (red), CH_2_ (blue), CH_3_ (magenta), Water (cyan), A4-153 (filled purple), A4-198 (filled blue), and Total (black).

**Figure 9:**
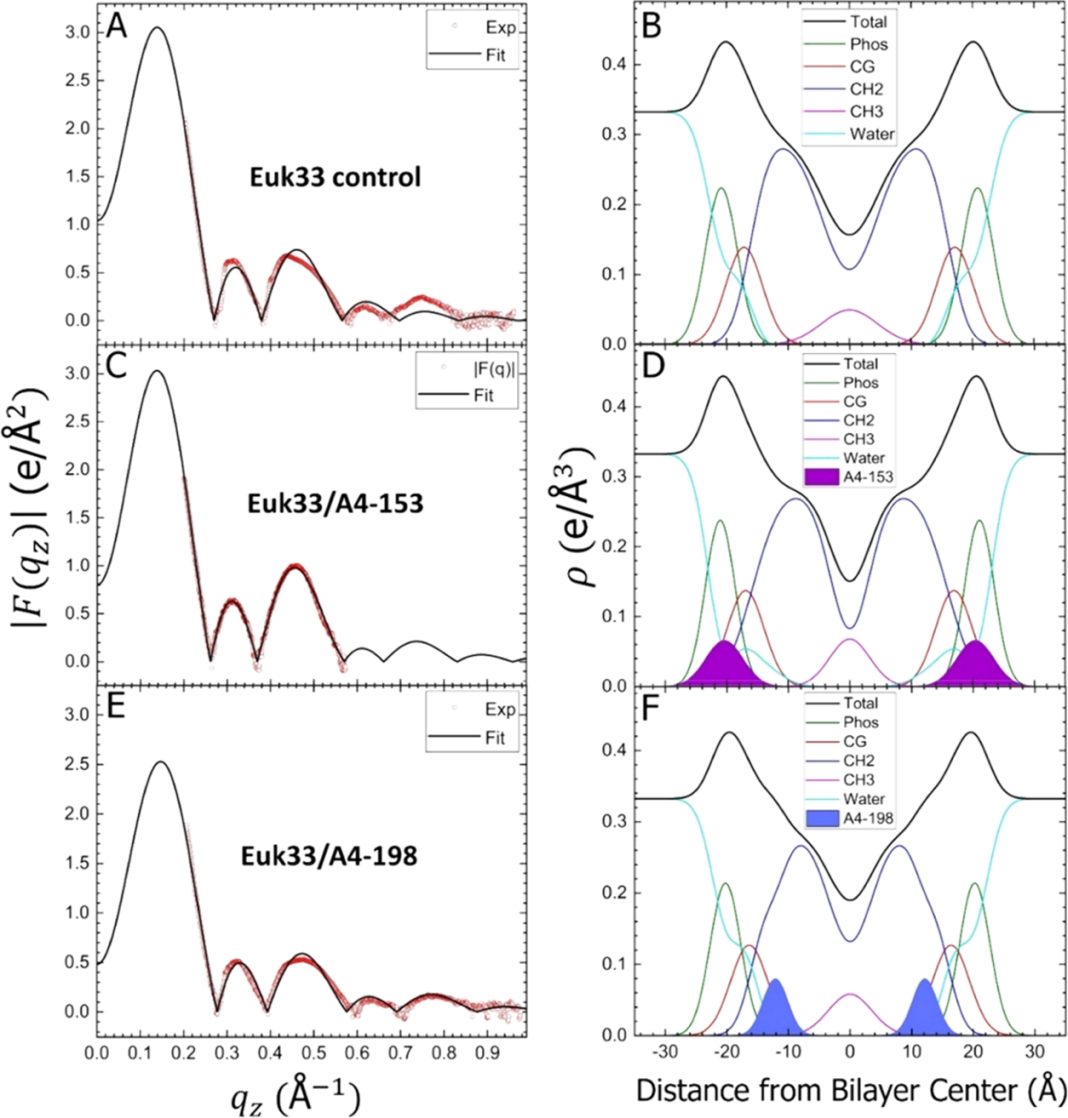
Form factor and EDP results for Euk33 membranes. Form factors: (A) Euk33 control, without peptide, (C) Euk33/A4-153 (75:1 molar ratio), and (E) Euk33/A4-198 (75:1). EDPs: (B) Control, (D) Euk33/A4-153 (75:1), and (F) Euk33/A4-198 (75:1). Component group and peptide colors as in Figure 8.

The *K*_C_ results are needed to obtain structural results.^25^Figures 8 and 9 show the form factors and electron density profiles (EDPs) obtained by fitting the XDS data with the scattering density profile (SDP) program, which takes into account the volumes of the lipid and peptides and component groups in the bilayer. As shown in Figures 8 and 9(A,C,E), there was an excellent fit of the SDP bilayer model to the XDS form factor data. The resulting EDPs shown in Figures 8 and 9(B,D,F) are typical of fully hydrated membranes. The component groups in the EDPs are Phos, phosphate plus outer headgroup; CG, carbonyl/glycerol; CH_2_, methylene hydrocarbon region which also contains CH groups; CH_3_, terminal methyl group; Water, which fills in the volumes around the other groups so that the total volume probabilities sum to one; and Total, which is the sum of all of the component groups. The reduced chi-squared values obtained during the SDP fit were lower when fitting A4-153 in the headgroup region with extension into the aqueous phase in G(−)IM, while A4-198 located in the upper hydrocarbon region. Similarly, in Euk33, A4-153 located in the bulk and in the outer headgroup region, while A4-198 located in the upper hydrocarbon region. Area and thickness results are summarized in Table 3, where 2*D*_C_ is the hydrocarbon thickness and *D*_HH_ is the distance between phosphate groups. Interestingly, for G(−) IM LMM, the area/lipid decreases with A4-153, while it increases for A4-198, which may be related to the peptides’ respective positions in the bilayer. For Euk33 LMM, there was only a small increase in area for A4-153, and a much larger increase for A4-198.

**Table 3:** Summary of Structural Results from XDS

sample	area/lipid (Å^2^) (±1)	2*D*_C_ (Å) (±0.5)	*D*_HH_ (Å) (±0.5)
G(−)/control	70.8	29.1	39.2
G(−)/153	69.9	29.5	39.9
G(−)/198	80.9	26.6	35.5
Euk33/control	64.2	32.0	40.3
Euk33/153	65.0	31.7	41.4
Euk33/198	74.0	29.7	38.9

### 3.6 Neutron Reflectivity (NR)

**Figure 10:**
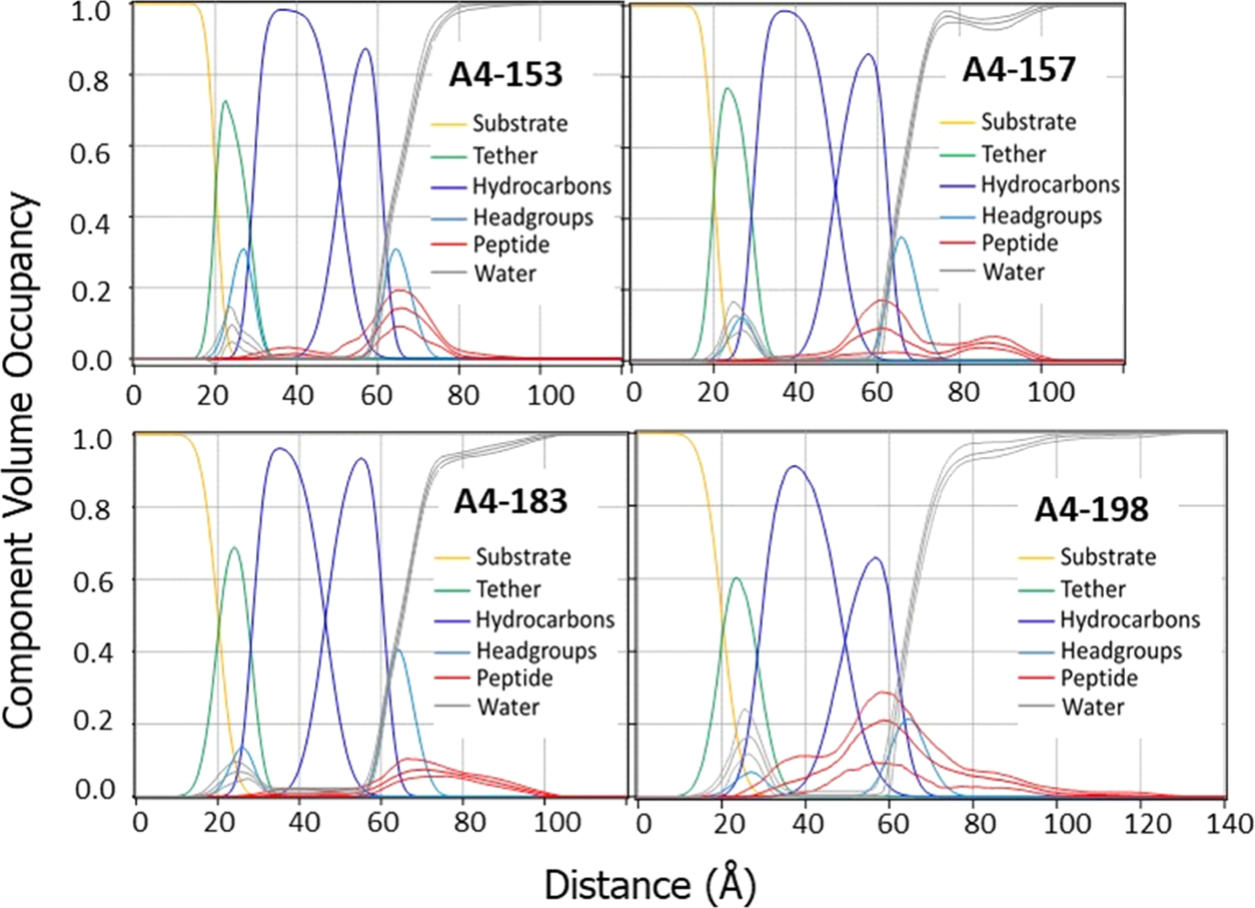
SPLUNC1-derived peptides in a single tethered bilayer of G(−)IM LMM. Component volumes: substrate (gold), tether (green), hydrocarbons (blue), headgroups (cyan), water (gray), and peptide (red). The pink lines represent the 68% confidence limit of the fit to the peptide data.

NR was employed to determine the location of each of the SPLUNC1-derived peptides in a single tethered bilayer of G(−)IM model membrane, as shown in Figure 10. While XDS also locates the position of the peptide in a lipid bilayer, NR is more accurate due to the higher contrast between peptide and lipid and the ability to change the solvent contrast. The red envelope in each of the graphs represents the peptide’s location with 68% confidence limits. As shown, A4-153 locates in the headgroup and partially in the bulk (aqueous phase), with partial penetration into the hydrocarbon region in G(−)IM LMM. A4-157 locates in the bulk and upper hydrocarbon regions about equally, and a smaller amount in the headgroup region. A4-183 locates primarily in the bulk and headgroup regions. A4-198 locates primarily in the hydrocarbon region but also in the headgroup and bulk. These locations are quantitatively summarized in Table 4; here, the component fractions add to 1.0 within standard deviations.

**Table 4:** Peptide Partitioning into Component Volumes in NR

	sample			
parameter	A4-153	A4-198	A4-183	A4-157
fraction of protein in hydrocarbons	0.20 ± 0.10	0.51 ± 0.08	0.14 ± 0.08	0.38 ± 0.09
fraction of protein in outer headgroups	0.50 ± 0.10	0.23 ± 0.07	0.26 ± 0.06	0.21 ± 0.09
fraction of protein in bulk solvent	0.25 ± 0.05	0.27 ± 0.06	0.59 ± 0.09	0.40 ± 0.10

## 4 Discussion

The SPLUNC1 peptides in this work all derive from A4S, which has been identified as a key peptide on the SPLUNC1 protein that has antimicrobial activity [2]. A4-153 had the smallest average MIC value compared to the other SPLUNC1-derived peptides and human LL-37. Out of the 13 colistin-susceptible substrains of *K. pneumoniae* that were analyzed, seven showed A4-153 to be the most effective at inhibiting biofilm formation at its MIC value of ∼16 μM (Figure 4). Moreover, at ∼16 μM, A4-153 was the most effective at inhibiting biofilm formation for eight out of the 11 colistin-resistant substrains, in addition to being effective against all 13 colistin-sensitive strains. At both lower and higher concentrations, the superiority of A4-153 in preventing biofilm formation was not as obvious. A possible cause for this is that peptide aggregation or experimental technique resulted in some absorbance values rising above the control value. While biofilm results are essential for fighting internal bacterial infections, the planktonic MIC values were experimentally robust. The MIC of A4-153 is ∼16 μM, which is not as low as that of colistin in the colistin-sensitive substrains (<2 μM), but colistin has been studied for decades, and its toxicity has been shown to be an issue in humans.^32^

Among the SPLUNC1 A4S derivatives that display antimicrobial properties, the toxicity data in Figure 5 show that A4-153 has comparable toxicity to control at low concentration (8μM) in murine RAW 264.7 cells (Figure 5A), while A4-183 has the greatest cytotoxicity at high concentrations in RAW 264.7 cells, 3T3 fibroblast cells, and HBE cells(Figures 5A,B and S3). But more important than cytotoxicity is the therapeutic index (TI), which is the concentration at which ≥90% of eukaryotic cells are killed divided by the concentration at which ≥90% of bacteria are killed. For A4-153, this TI value is an estimated ≈ 128 μM/16 μM ≈ 8. TI for A4-157 is an estimated 128 μM/24 μM ≈ 5, and the TI for A4-183 is an estimated 64 μM/20 μM ≈ 3.

**Figure 11:**
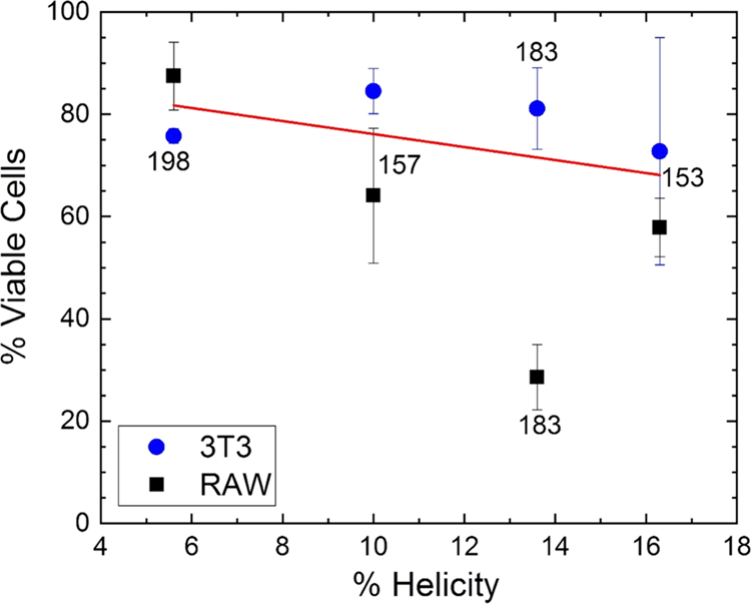
Toxicity vs helicity results in Euk33 LMMs for SPLUNC1-derived AMPs. Viable cell count for two types of eukaryotic cells at 32 μM AMP concentration was used as a measure of toxicity. A4-183 in RAW cells was considered to be an outlier and was not included in the linear fit.

**Figure 12:**
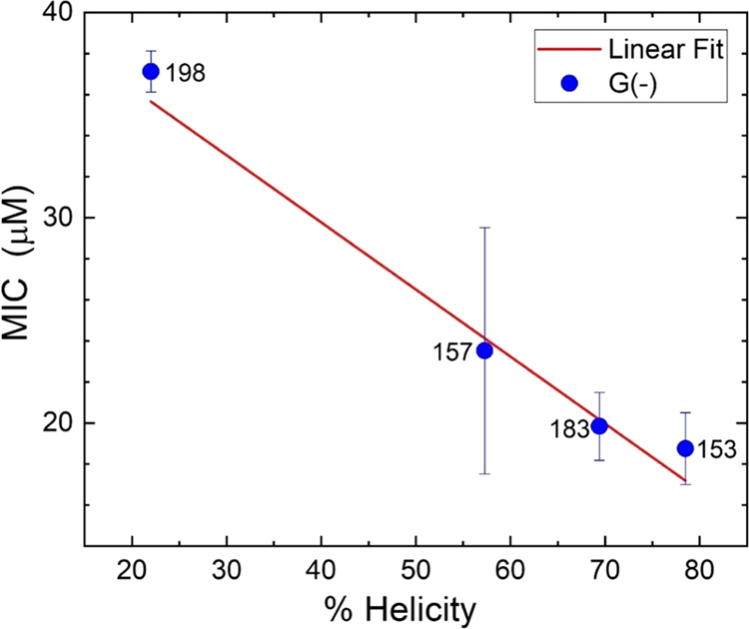
Efficacy vs helicity results in G(−) LMMs for SPLUNC1-derived AMPs. All data points were included in the linear fit.

A4-153’s primary structure may contribute to its success over the other three A4S derivatives. A4-153 was designed by substituting the N-terminal phenylalanine in A4-157 with leucine, and the penultimate C-terminal valine with isoleucine. These two small changes increased the hydrophobicity H but hardly changed the hydrophobic moment μH, so the μH/H is only slightly lower for A4-153 than for A4-157. Both peptides are helical, with A4-153 more helical at 30:1 lipid/peptide molar ratio. This suggests that helical content is an important property for predicting AMP activity as has been noted for other AMPs.^33,34^ However, we published previously that this is not always the case. For example, the D8 form of WLBU2, containing eight valines as the D-enantiomer, displayed a random coil structure in G(−) LMMs, unlike WLBU2’s mainly helical structure, yet both AMPs had equal efficacy at killing G(−) bacteria.^35,36^ Other efficient AMPs rely on different secondary structures, such as β-sheets.^37^ The toxicity of A4-153 at 32 μM (Figure 5) was linearly correlated with the highest helicity (Figure 11), where we plot the highest helicity values from the CD tables in the S.I. for each A4S derivative vs their percent toxicities measured at 32 μM. As shown, there is a linear relationship between percent helicity and percent toxicity (decrease of viable cells percentage) when 3T3 and RAW 264.7 cell types are averaged. In addition, the lowest MIC values were linearly correlated with the highest helicities (Figure 12). These efficacy results agree with previously obtained experimental results using other peptides, which showed a correlation between higher helicity and higher bacterial killing efficacy.^38−45^

As for the location of the A4S peptides in the membrane, we demonstrate that the more successful peptides are located in the headgroup and/or bulk region (see Figures 8–10 and Table 4). A4-153 is able to kill bacteria with very little penetration into bacterial membranes, while A4-198 is ineffective and is located primarily in the hydrocarbon region. As shown in Table 3, the area/lipid decreases slightly with A4-153 in the G(−) LMM. Such condensation of the headgroup suggests that electrostatics and/or hydrogen bonding may shrink the headgroup, opening up a defect region between adjacent lipids. This did not occur with A4-198; in fact, there was a large areal increase, as A4-198 located more deeply in the membrane, perhaps blocking a water channel, thus ineffective at bacterial killing. As for toxicity, we used a second LMM that mimics a white blood cell membrane with 33 mole % cholesterol. A4-198 penetrates more deeply than A4-153 in Euk33 LMM (Figure 9), yet it is less toxic, which contradicts a previous study that found deeper penetration of another AMP into the hydrocarbon region in higher cytotoxicity in red blood cells.^33^

Besides structural results, XDS also yields material property results. While there were four peptides in the microbiological part of this work, only A4-153 and A4-198 were probed using XDS due to X-ray synchrotron time constraints. For G(−) LMM, there was little difference in bending modulus between A4-153 and A4-198, suggesting that membrane rigidity is not important in bacterial killing efficacy. For chain order, there was a dramatic difference, in that A4-153 had more ordered chains. For toxicity, both peptides softened and disordered chains, although this was more pronounced with A4-198. Thus, a certain rigidity and chain order may be required for eukaryotic membrane toxicity.

Our goal has always been to develop novel antimicrobials that exhibit negligible toxicity while maintaining potent antimicrobial activity to overcome antibiotic-resistant bacterial infections. Results from this study suggest A4-153 could be a candidate if lower toxicity can be shown in additional cell types and animal experiments. However, as presented in our manuscript, the critical knowledge obtained from this study provides insightful and helpful information for continually improving the peptide design. Thus, we will expand A4-153-related studies in parallel with the additional design of new AMPs based on the presented data to determine the best peptide option for clinical applications.

## 5 Conclusions

A4-153 is the most successful AMP compared to the three other derivatives of A4S, as it has the smallest MIC value in these G(−) bacteria, and it is the most effective at inhibiting biofilm formation for most of the *K. pneumoniae* substrains. The CD data reveal that it also has the greatest α-helical character. The NR and XDS results show that A4-153 locates primarily in the headgroup region and shrinks the area per lipid. This may be important in opening up a defect in the membrane to allow for release of water and ions from the bacteria. Furthermore, A4-198, which was designed as a similar peptide scrambled to helix formation, displayed the smallest helical character, the lowest bacterial killing effectiveness, and the lowest toxicity. Toxicity in eukaryotic LMMs is directly correlated with a headgroup location of A4 peptides and a greater α-helical content. Our elasticity results suggest that bacterial killing efficacy is uncorrelated with membrane stiffness but that ordered lipid chains are required. Eukaryotic membrane toxicity is correlated with stiffer membranes and more ordered chains. As for rational design, there was a clear correlation between μH or μH/H ratio and bacterial killing efficiency when comparing the scrambled peptide A4-198 with the other three peptides. However, in the remaining mostly helical peptides, we found no clear correlation between bacterial killing efficiency and length of peptide, overall charge, μH or μH/H ratio.

## Supporting Information Available

Toxicity standard curves for the data in Figure 5, additional toxicity studies for two cell types HBE cells (Figure S3) and THP-1 cells (Figure S4), CD results (Tables S1–S8), an example of LAXS (Figure S5A) and WAXS (Figure S5B), XDS for A4-153 in G(+) LMMs at 100:1 mole ratio, and HPLC/MS data from Genscript (Figures S6–S9) (PDF)

## Abbreviations

AMP: antimicrobial peptide
ASL: airway surface liquid
CHESS: Cornell High Energy Synchrotron Source
CD: circular dichroism
EDPs: electron density profiles
Euk33: eukaryotic with 33 mole% cholesterol
LMMs: lipid model membranes
G(−): Gram-negative
MCA: mucociliary apparatus
MDR: multi-drug-resistant
MIC: minimum inhibitory concentration
MRE: mean residue ellipticity
NR: neutron reflectometry
SPLUNC1: short palate lung and nasal epithelial clone 1
ULVs: unilamellar vesicles
