# Correction to “Lung SPLUNC1 Peptide Derivatives in the Lipid Membrane Headgroup Kill Gram-Negative Planktonic and Biofilm Bacteria”

**DOI:** 10.1021/acs.biomac.4c00501

**Published:** 2024-04-19

**Authors:** Tanvi Jakkampudi, Qiao Lin, Saheli Mitra, Aishwarya Vijai, Weiheng Qin, Ann Kang, Jespar Chen, Emma Ryan, Runxuan Wang, Yuqi Gong, Frank Heinrich, Junming Song, Yuan-Pu Peter Di, Stephanie Tristram-Nagle

There is an error in Table 1. Please replace the peptide sequences with the following:

A4-153 LKKFFKKVKGWVGGVWGKVKSVIK 24.

A4-157 FKKFFKKVKGWVGGVWGKVKSVVK 24.

A4-183 FKKFLKKFKGWLGGLWGK 18.

A4-198 GVKKKWKKKLGLTLWLKISGVVLG 24.

The underlined amino acids were missing in Table 1. A4-183 is correct.

