# Supplementary material for: Lung SPLUNC1 Peptide Derivatives in the Lipid Membrane Headgroup Kill Gram-Negative Planktonic and Biofilm Bacteria
Source: Biomacromolecules. 2023 May 24;24(6):2804–15. doi: 10.1021/acs.biomac.3c00218 (PMC10265666; doi:10.1021/acs.biomac.3c00218)
Supplement: Supplementary file 1 — bm3c00218_si_001.pdf [file bm3c00218_si_001.pdf]

SUPPLEMENTARY INFORMATION

Lung SPLUNC1 Peptide Derivatives in the Lipid Membrane Headgroup Kill Gram-Negative Planktonic and Biofilm Bacteria

Tanvi Jakkampudi[1], Qiao Lin[2], Saheli Mitra[1], Aishwarya Vijai[1], Weiheng Qin[1], Ann Kang[1], Jespar Chen[1], Emma Ryan[1], Runxuan Wang[1], Yuqi Gong[1], Frank Heinrich[1,3], Junming Song[2], Yuan-Pu (Peter) Di[*2], Stephanie Tristram-Nagle[*1]

[1]Biological Physics Group, Physics Department, Carnegie Mellon University, Pittsburgh, PA

[2]Department of Environmental and Occupational Health, University of Pittsburgh, Pittsburgh, PA

[3]Center for Neutron Research, National Institute of Standards and Technology, Gaithersburg, MD

*Co-corresponding authors

Rationale for peptide design

The design of the four new A4S peptides was based on the previously published SPLUNC1 peptide A4-Short. We maintained the same 24 amino acid length in three of the four peptides, except for A4-183, but varied their charges and hydrophobic moments to modulate their antimicrobial activities and toxicities. We increased two positively charged amino acids to increase the potential antimicrobial activity of A4-153, and replaced two tryptopahan (W) residues with phenylalanine (F) to reduce host toxicity. A4-157 is similar to A4-153 except for a substitute of the first amino acid leucine (L) with phenylalanine (F), and a switch of the 23rd amino acid isoleucine (I) with valine (V), resulting in a symmetrical helical position. A4-183 is a shortened peptide of A4-157, with only 18 amino acids in length, by deleting the last six amino acids and

making two valine (V) to leucine (L) substitutions. These changes make a perfectly amphipathic structure with a symmetrical amino acid arrangement in a helical wheel diagram. Finally, A4-198 is a peptide with a similar amino acid composition and length as A4-153 and A4-157, but with a rearranged amino acid sequence to remove the amphpathic structure and significantly reduce hydrophobic moments intentionally, resulting in no helical structure and minimal toxicity, but with diminished antimicrobial activity.

Standard Curves for Toxicity Measurements shown in Fig. 5 in main paper

Figs. S1 and S2 show the standard curves for murine RAW 264.7 cells and murine 3T3 fibroblasts cells collected at 37 ^{o}C. These standard curves were created using known viable cell counts and measuring them with a Biotek Epoch2 Microplate Reader at λ=450 nm to obtain corresponding optical density (OD) values. The OD values were obtained in duplicate, so the average values were plotted. Upon fitting the data to yield minimum R^2 values, the best lines of fit were used to correlate experimentally obtained OD values to viable cell counts.

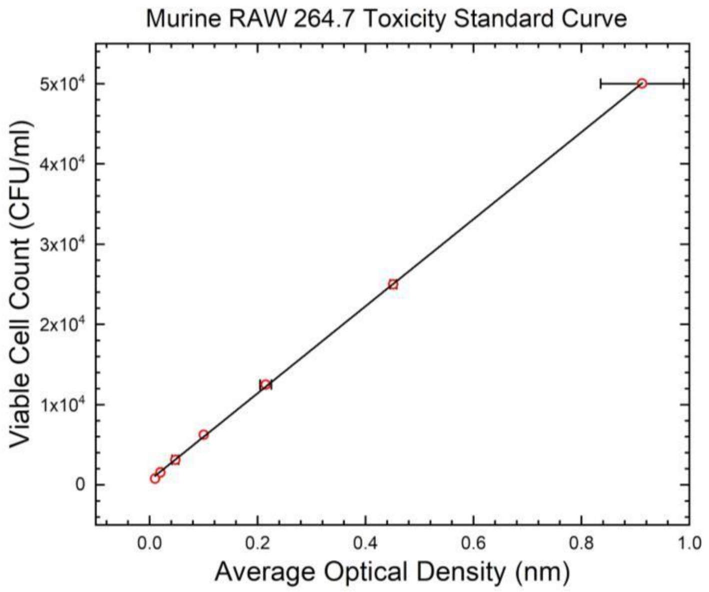

Figure S1. Toxicity standard curve for murine Raw 264.7 cells. Linear fit: Y = 54250.9X + 557.1.

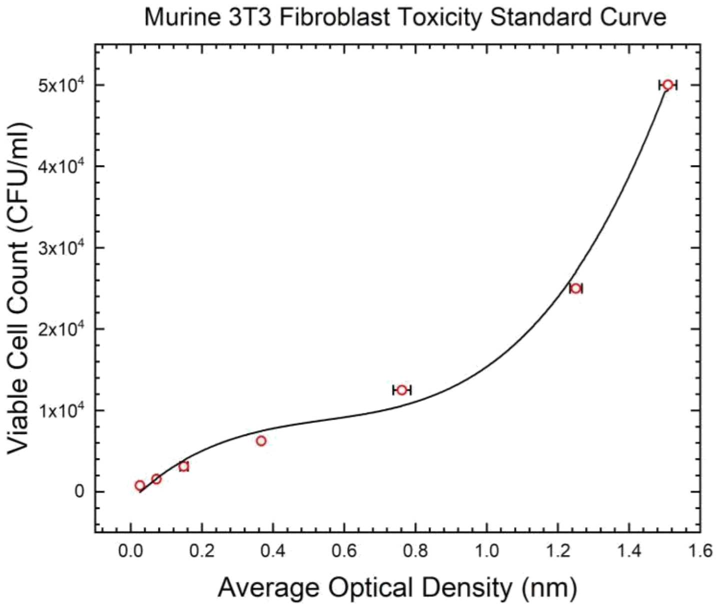

Figure S2. Toxicity standard curve for murine 3T3 fibroblast cells. Polynomial Fit: Y = 39058.8X^3 – 64430.1X^2 + 41654.7X – 1106.5.

Additional toxicity studies at 16 hours incubation with peptides

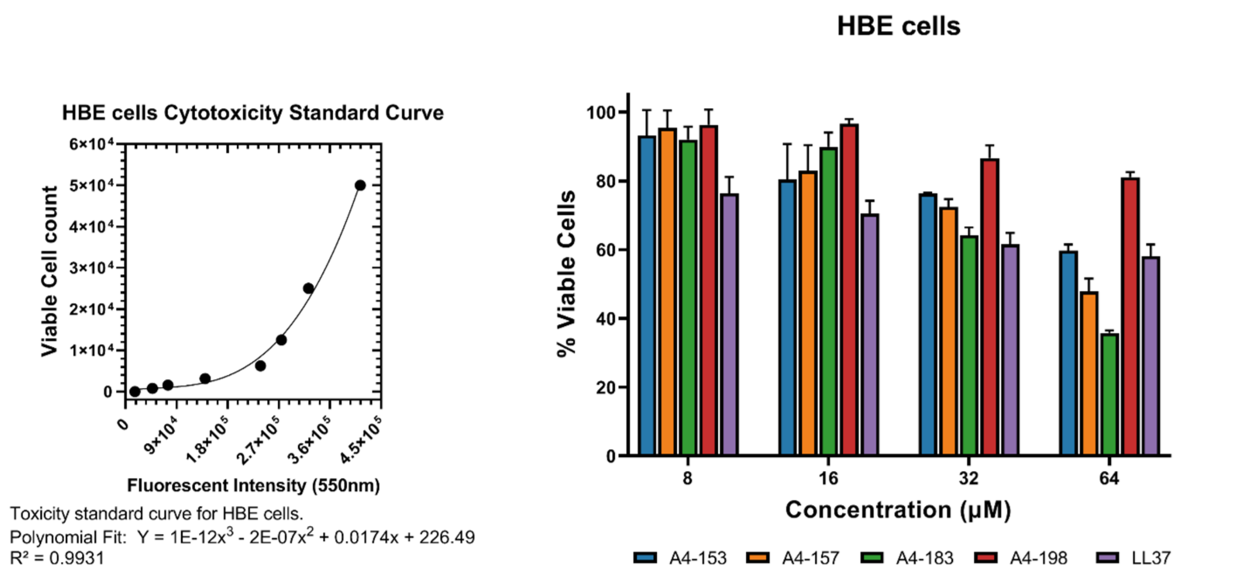

Figure S3.

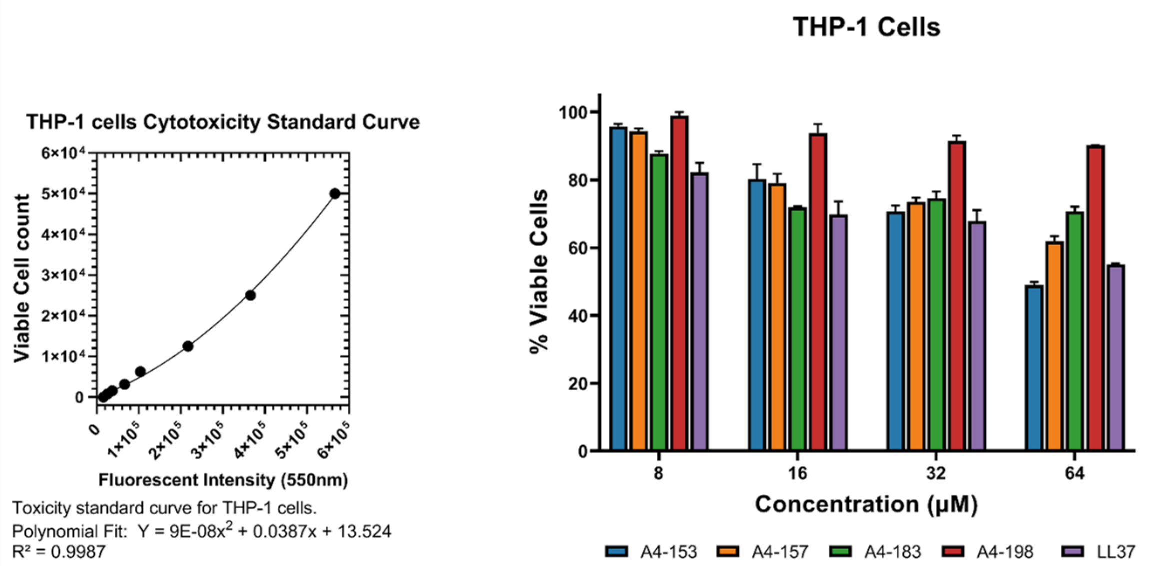

Figure S4.

Two additional cell lines, HBE and THP1 cells (Figs. S3 and S4), confirmed the general trend of 3T3 and RAW264.7 cells (Fig. 5), but A4-183 was less toxic in THP-1 cells than in the other cell types.

CD Results

The following tables show secondary structure results for A4-153, A4-157, A4-183, and A4-198 in two different lipid model membranes, G(-) IM and Euk33. These tables provide more detail about the four structural motifs, α-helix, β-sheet, β-turn and random coil than in the main paper. For each table, R^2 indicates goodness of fit. Therefore, these structural results from CD spectroscopy give an indication of % helix as well as the other three motifs, which can be compared to their antimicrobial activity. As stated in the main paper, A4-153 had the highest helical content in both lipid model membranes, and also was by far the most effective AMP in the planktonic and biofilm experiments with *K. pneumoniae* bacterial strains (Fig. 2,3 in the main paper.

A4-153 CD Results

Table S1. A4-153 CD results of secondary structure in G(-) IM mimics

G(-) IM/A4-153 Molar ratio	α-helix (%)	β-sheet (%)	β-turn (%)	Random (%)	R^2
0:1	6.4	34.2	1.7	57.7	0.98
1:1	11.9	32.4	4.0	51.7	0.88
5:1	10.2	26.9	0	62.9	0.98
10:1	14.8	17.7	5.0	62.5	0.97
15:1	41.9	41.2	1.3	15.6	0.94
20:1	78.3	8.0	13.7	0	0.97
30:1	78.5	10.2	6.7	4.6	0.93
50:1	77.4	13.6	9.0	0	0.77
70:1	64.1	15.1	20.8	0	0.88

Table S2. A4-153 CD results of secondary structure in Euk33 mimics

Euk-33/A4-153 Molar ratio	α-helix (%)	β-sheet (%)	β-turn (%)	Random (%)	R^2
0:1	6.4	34.2	1.7	57.7	0.98
5:1	9.7	24.1	0	66.2	0.99
10:1	12.2	23.6	0	64.2	0.99
20:1	14.1	25.5	0	60.4	0.98
30:1	16.2	26.3	0	57.5	0.97
70:1	20.5	28.2	0	51.3	0.85

A4-157 CD Results

Table S3. A4-157 CD results of secondary structure in G(-) IM mimics

G(-) IM/A4-157 Molar ratio	α-helix (%)	β-sheet (%)	β-turn (%)	Random (%)	R^2
0:1	4.57	33.6	0	61.7	0.98
1:1	6.9	31.4	0	61.7	0.99
5:1	15.1	25.4	0	59.5	0.98
10:1	19.0	28.3	0.1	52.6	0.96
15:1	46.0	33.9	0	20.1	0.97
20:1	49.3	24.2	0	26.5	0.96
25:1	61.0	28.5	2.2	8.3	0.99
30:1	62.1	28.7	1.1	8.1	0.96
40:1	59.5	40.5	0	0	0.98
50:1	56.4	33.6	0	1.0	0.97
70:1	51.2	35.6	0	13.2	0.98

Table S4. A4-157 CD results of secondary structure in Euk33 mimics

Euk-33/A4-157 Molar ratio	α-helix (%)	β-sheet (%)	β-turn (%)	Random (%)	R^2
0:1	4.57	33.6	0	61.7	0.98
5:1	3.3	32.5	0	64.2	0.99
10:1	7.3	28.3	0	64.4	0.97
30:1	10.0	30.9	0	59.1	0.96
70:1	7.4	50.1	0	42.5	0.73
0:1	4.57	33.6	0	61.7	0.98

A4-183 CD Results

Table S5. A4-183 CD results of secondary structure in G(-) IM mimics

G(-) IM/A4-183 Molar ratio	α-helix (%)	β-sheet (%)	β-turn (%)	Random (%)	R^2
0:1	3.1	31.0	0	65.9	0.97
1:1	4.7	31.2	0	64.1	0.98
5:1	22.6	18.2	0	59.2	0.96
10:1	44.4	14.9	11.6	29.1	0.93
15:1	63.0	14.1	22.5	0.4	0.95
20:1	66.7	8.8	12.1	12.4	0.99
25:1	64.9	12.5	10.9	11.7	0.99
30:1	69.4	8.7	8.9	13.0	0.95
50:1	63.6	9.1	9.1	18.2	0.92

Table S6. A4-183 CD results of secondary structure in Euk33 mimics

Euk-33/A4-183 Molar ratio	α-helix (%)	β-sheet (%)	β-turn (%)	Random (%)	R^2
0:1	3.1	31.0	0	65.9	0.97
5:1	8.6	27.3	0	64.1	0.97
10:1	13.0	19.2	0	67.8	0.96
20:1	10.4	28.4	0	61.2	0.98
30:1	13.6	26.4	0	60.0	0.97
70:1	12.2	50.4	0	37.4	0.83

A4-198 CD Results

Table S7. A4-198 CD results of secondary structure in G(-) IM mimics

G(-) IM/A4-198 Molar ratio	α-helix (%)	β-sheet (%)	β-turn (%)	Random (%)	R^2
0:1	2.9	56.7	4.5	35.9	0.99
1:1	2.6	55.9	8.8	32.7	0.99
5:1	22.0	24.4	12.6	41.0	0.98
10:1	15.5	32.0	10.4	42.11	0.97
15:1	9.1	48.1	30.7	12.1	0.99
20:1	4.6	44.5	20.2	30.7	0.98
30:1	0.2	44.5	20.4	34.9	0.94
50:1	0.2	46.4	15.8	37.6	0.96
70:1	2.0	46.2	14.7	37.1	0.98

Table S8. A4-198 CD results of secondary structure in Euk33 mimics

Euk-33/A4-198 Molar ratio	α-helix (%)	β-sheet (%)	β-turn (%)	Random (%)	R^2
0:1	2.9	56.7	4.5	35.9	0.99
5:1	5.7	53.6	6.0	34.7	0.99
10:1	5.9	51.9	5.2	37.0	0.99
30:1	6.2	49.4	5.7	38.7	0.97
70:1	10.3	47.9	0	41.8	0.98

XDS Results

Fig. S5 shows LAXS and WAXS results for A4-153 in G(-)IM mimic with 100:1 lipid:peptide molar ratio, as an example of all lipid:peptide molar ratios, which were similar. The LAXS intensity data such as the example shown in Fig. S5A were used to calculate the bending modulus as a function of protein concentration (Fig 8A,B in the main paper). These data were also used to obtain the form factors (Figs. 9,10 (A,C,E)). The Fourier transform of these form factors yielded the electron density profiles provided in the main paper (Figs. 9,10 (B,D,F). The WAXS intensity data such as shown in Fig. S5B, were used to obtain the chain order parameter, S_{xray}, shown in Figs. (8C,D) as described in Materials and Methods.

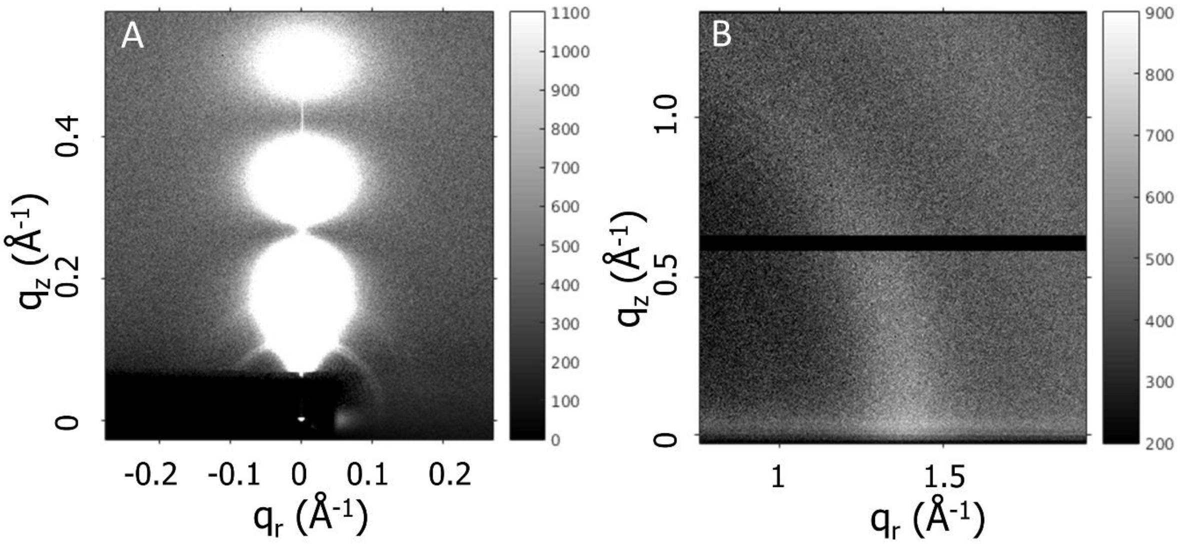

Figure S5. XDS data obtained at CHESS. Results of 500:1 G(-)/A4-153 at 37℃. **A.** Low angle x-ray scattering (LAXS). A lamellar Bragg order is visible for h=2 through the semi-transparent Molybdenum beam stop (dark rectangle near bottom). The sample is fully hydrated with a D-spacing of 165 Å. Three lobes of diffuse x-ray scattering result from fluctuations in the oriented stack of membranes at high hydration. **B.** Wide angle x-ray scattering (WAXS). The chain correlation is the intensity centered at $q_r \cong 1.4$ Å^{-1} which corresponds to ≅4.5 Å d-spacing. Light grey indicates positive intensity values (see color bars). Black line contains no intensity information and was removed during data analysis.

Sample Name: A4-153
Sample ID: U347WEL230-1
Time Processed : 20:58:57
Month-Day-Year Processed : 12/26/2019

Pump A : 0.065% trifluoroacetic in 100% water (v/v)
Pump B : 0.05% trifluoroacetic in 100% acetonitrile (v/v)
Total Flow:1 ml/min
Wavelength:220 nm
<<LC Time Program>>

Time	Module	Command	Value
0.01	Pumps	Pump B Conc.	5
25.00	Pumps	Pump B Conc.	65
25.01	Pumps	Pump B Conc.	95
27.00	Pumps	Pump B Conc.	95
27.01	Pumps	Pump B Conc.	5
32.00	Pumps	Pump B Conc.	5
32.01	Controller	Stop	

<<Column Performance>>
<Detector A>
Column :Inertsil ODS-3 4.6 x 250 mm
Equipment:GK12010012

<Chromatogram>

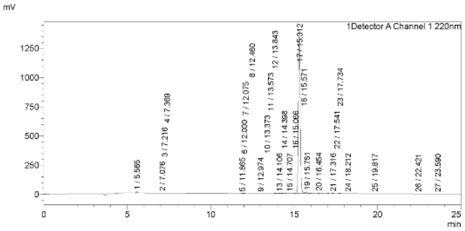

<Peak Table>

Detector A Channel 1 220nm

Peak#	Ret. Time	Area	Height	Area%
1	5.565	66664	11701	0.601
2	7.076	2527	296	0.023
3	7.216	2326	354	0.021
4	7.369	3357	332	0.030
5	11.865	2758	225	0.025
6	12.000	1098	233	0.010
7	12.075	1863	216	0.017
8	12.460	3501	387	0.032
9	12.974	1018	138	0.009
10	13.373	1956	196	0.018
11	13.573	356	64	0.003
12	13.843	11485	1621	0.104
13	14.106	13708	1405	0.124
14	14.398	32569	2884	0.294
15	14.707	6474	721	0.058

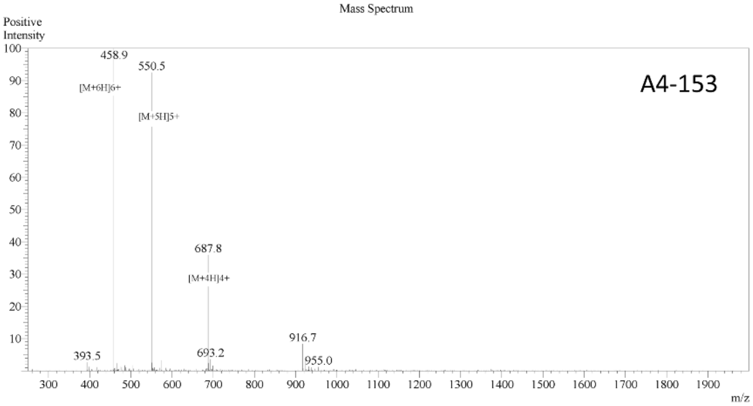

Sample Information
Acquired by : Gary
Month-Day Processed : 12/25/19
Time Processed : 18:03:58
Injection Volume : 0.3
Sample Name : A4-153
Sample ID : U347WEL230-1
Theoretical MW : 2747.43
Observed MW : 2747.4

Interface :ESI
Nebulizing Gas Flow :1.5L/min
CDL Temp :250
Block Temp :200

Equipment : GK11010007
Interface Bias : +4.5 kV
Drying Gas Flow :5 L/min
T.Flow :0.2 ml/min
B.conc :50%H2O/50%MeOH

Peak#	Ret. Time	Area	Height	Area%
16	15.066	11115	1401	0.100
17	15.312	10711544	1392932	96.645
18	15.571	47987	9366	0.433
19	15.751	82047	10844	0.740
20	16.454	17463	2983	0.158
21	17.316	21336	1972	0.193
22	17.541	1614	283	0.015
23	17.734	27110	3251	0.245
24	18.212	1348	237	0.012
25	19.817	1852	165	0.017
26	22.421	3150	372	0.028
27	23.590	5159	632	0.047
Total		11083386	1445211	100.000

Figure S6. High pressure liquid chromatography (HPLC, left) and Mass spectroscopy (MS, right) of A4-153. Data provided by Genscript.

Sample Name : A4-157
Sample ID : U347WEL230-3
Time Processed :20:38:09
Month-Day-Year Processed :01/07/2020

Pump A : 0.065% trifluoroacetic in 100% water (v/v)
Pump B : 0.05% trifluoroacetic in 100% acetonitrile (v/v)
Total Flow:1 ml/min
Wavelength:220 nm
<<LC Time Program>>

Time	Module	Command	Value
0.01	Pumps	Solvent B Conc.	5
25.00	Pumps	Solvent B Conc.	65
25.01	Pumps	Solvent B Conc.	95
27.00	Pumps	Solvent B Conc.	95
27.01	Pumps	Solvent B Conc.	5
35.00	Pumps	Solvent B Conc.	5
35.01	Controller	Stop	

<<Column Performance>>
<Detector A>
Column : Inertsil ODS-3 4.6 x 250 mm
Equipment:ZJ19010140

<Chromatogram>

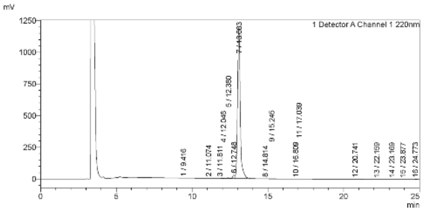

<Peak Table>

Detector A Channel 1 220nm

Peak#	Ret. Time	Area	Height	Area%
1	9.416	1563	92	0.010
2	11.074	1099	94	0.007
3	11.811	8836	938	0.058
4	12.046	37642	3139	0.247
5	12.380	58477	5472	0.383
6	12.748	258544	27639	1.695
7	13.083	14846350	1184603	97.357
8	14.814	1174	168	0.008
9	15.245	10528	712	0.069
10	16.809	1215	187	0.008
11	17.039	3483	440	0.023
12	20.741	1844	194	0.012
13	22.159	1529	117	0.010
14	23.169	1233	124	0.008
15	23.877	2086	148	0.014
16	24.773	13839	1335	0.091
Total		15249443	1225403	100.000

Mass Spectrum

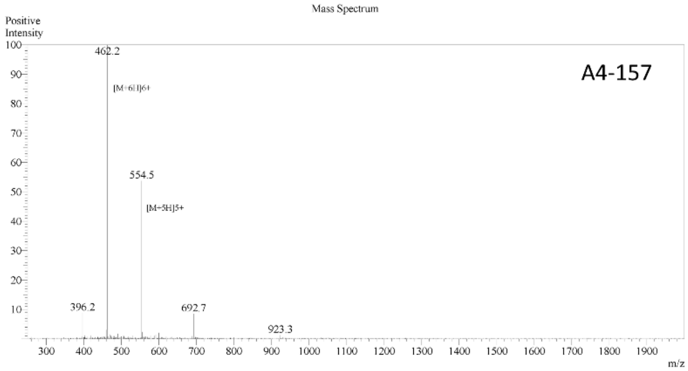

Sample Information
Acquired by : Gary
Month-Day Processed : 01/06/20
Time Processed : 18:53:43
Injection Volume : 0.3
Sample Name : A4-157
Sample ID : U347WEL230-3
Theoretical MW : 2767.42
Observed MW : 2767.2

Interface :ESI
Nebulizing Gas Flow :1.5L/min
CDL Temp :250
Block Temp :200

Equipment : GK11010007
Interface Bias : +4.5 kV
Drying Gas Flow :5 L/min
T.Flow :0.2 ml/min
B.conc :50%H2O/50%MeOH

Figure S7. HPLC (left) and MS (right) of A4-157. Data provided by Genscript.

Sample Name:A4-183
Sample ID :U347WEL230-5
Time Processed: 17:56:47
Month-Day-Year Processed :01/03/2020

Pump A : 0.065% trifluoroacetic in 100% water (v/v)
Pump B : 0.05% trifluoroacetic in 100% acetonitrile (v/v)
Total Flow:1 ml/min
Wavelength:220 nm
<<LC Time Program>>

Time	Module	Command	Value
0.01	Pumps	Pump A B.Conc	15
25.00	Pumps	Pump A B.Conc	75
25.01	Pumps	Pump A B.Conc	95
32.00	Pumps	Pump A B.Conc	95
32.01	Pumps	Pump A B.Conc	5
40.00	Pumps	Pump A B.Conc	5
40.01	Controller	Stop	

<<Column Performance>>
<Detector A>
Column : AlltimaTM C18 4.6 x 250 mm
Equipment:ZJ19010324

<Chromatogram>

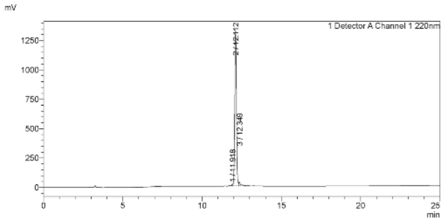

<Peak Table>

Detector A Channel 1 220nm

Peak#	Ret. Time	Area	Height	Area%
1	11.918	107284	15510	1.037
2	12.112	9884941	1324590	95.521
3	12.349	356242	33244	3.442
Total		10348468	1373345	100.000

Mass Spectrum

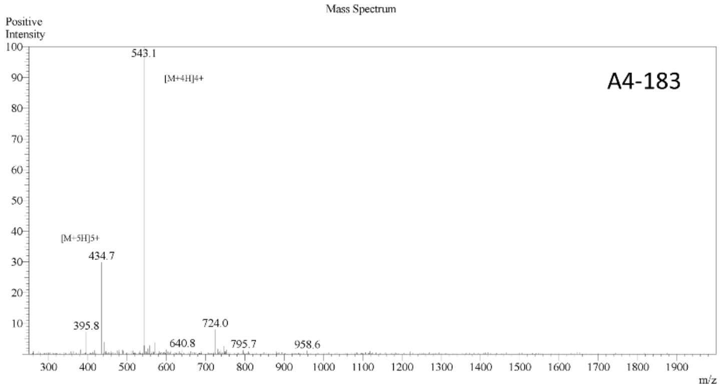

Sample Information
Acquired by : Gary
Month-Day Processed : 01/04/20
Time Processed : 17:11:30
Injection Volume : 0.3
Sample Name : A4-183
Sample ID : U347WEL230-5
Theoretical MW : 2168.68
Observed MW : 2168.4

Interface :ESI
Nebulizing Gas Flow :1.5L/min
CDL Temp :250
Block Temp :200

Equipment : GK11010007
Interface Bias : +4.5 kV
Drying Gas Flow :5 L/min
T.Flow :0.2 ml/min
B.conc :50%H2O/50%MeOH

Figure S8. HPLC (left) and MS (right) of A4-183. Data provided by Genscript.

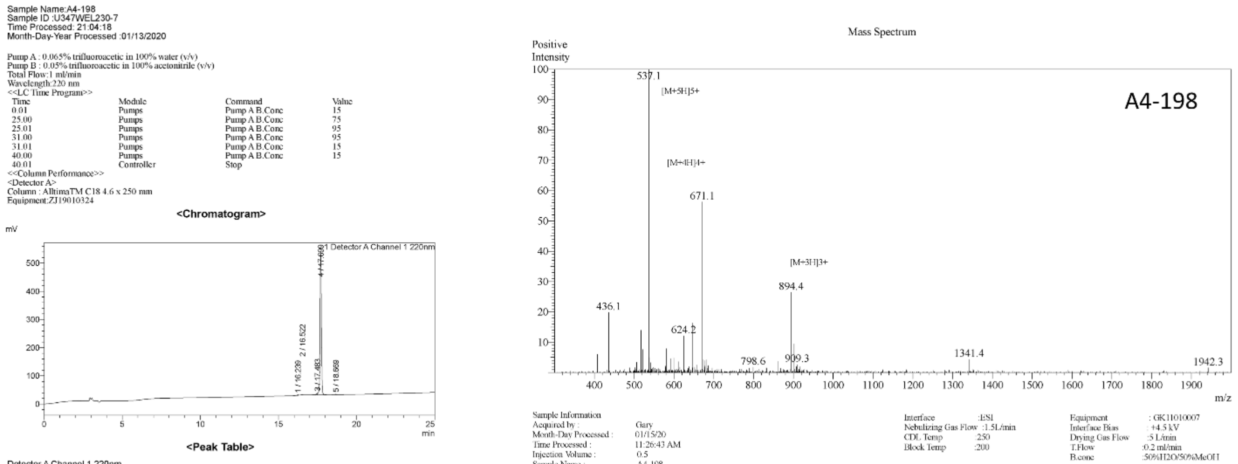

Peak#	Ret. Time	Area	Height	Area%
1	16.239	10307	2023	0.322
2	16.522	9659	1456	0.302
3	17.483	38009	5855	1.189
4	17.699	3124572	509837	97.716
5	18.669	15067	2309	0.471
Total		3197614	521480	100.000

Figure S9. HPLC (left) and MS (right) of A4-198. Data provided by Genscript.
